# PHGDH activation fuels glioblastoma progression and radioresistance via serine synthesis pathway

**DOI:** 10.1186/s13046-025-03361-3

**Published:** 2025-03-19

**Authors:** Xiaojin Liu, Bangxin Liu, Junwen Wang, Hongbin Liu, Jiasheng Wu, Yiwei Qi, Yuan Liu, Hongtao Zhu, Chaoxi Li, Liu Yang, Jian Song, Guojie Yao, Weidong Tian, Kai Zhao, Lin Han, Kai Shu, Suojun Zhang, Jianghong Man, Chao You, Haohao Huang, Ran Li

**Affiliations:** 1 Department of Neurosurgery, Tongji Hospital, Tongji Medical College, Huazhong University of Science and Technology, Wuhan, Hubei China; 2 Department of Neurosurgery, General Hospital of Central Theatre Command of People’s Liberation Army, Wuhan, Hubei China; 3 Wuhan University of Science and Technology, Wuhan, Hubei China; 4 Department of Neurosurgery, The First Affiliated Hospital of Shihezi University, Shihezi, China; 5 State Key Laboratory of Proteomics, National Center of Biomedical Analysis, Beijing, China; 6 General Hospital of Central Theater Command and Hubei Key Laboratory of Central Nervous System Tumor and Intervention, Wuhan, Hubei China

**Keywords:** PHGDH, Glioma stem cells, Serine synthesis pathway, MYC, Radioresistance

## Abstract

**Background:**

Glioma stem-like cells (GSCs) are key drivers of treatment resistance and recurrence in glioblastoma (GBM). Phosphoglycerate dehydrogenase (PHGDH), a crucial enzyme in the de novo serine synthesis pathway (SSP), is implicated in tumorigenesis and therapy resistance across various cancers. However, its specific role in GBM, particularly in radioresistance, remains poorly understood.

**Methods:**

In silico analysis of GBM patient data assessed SSP enrichment and PHGDH expression linked with tumor stemness. Comparative gene expression analysis focused on PHGDH in paired GBM specimens and GSCs. Genetic and pharmacological loss-of-function assays were performed in vitro and in vivo to evaluate PHGDH’s impact on GSC self-renewal and malignant progression. Comprehensive transcriptomic and metabolomic analyses, along with chromatin immunoprecipitation, mass spectrometry, and various other biochemical assays, were used to elucidate PHGDH-mediated mechanisms in GBM progression and radioresistance.

**Results:**

PHGDH expression is significantly elevated in GSCs, associated with aggressive glioma progression and poor clinical outcomes. PHGDH activation enhances GSC self-renewal by regulating redox homeostasis, facilitating one-carbon metabolism, and promoting DNA damage response via SSP activation. Importantly, MYC was identified as a crucial transcriptional regulator of PHGDH expression. Furthermore, genetic ablation or pharmacological inhibition of PHGDH markedly reduced tumor growth and increased tumor sensitivity to radiotherapy, thereby improving survival outcomes in orthotopic GSC-derived and patient-derived GBM xenograft models.

**Conclusions:**

This study underscores the pivotal role of MYC-mediated PHGDH activation in driving GSC malignant progression and radioresistance in GBM. Targeting PHGDH presents a promising approach to enhance radiotherapy efficacy in GBM patients.

**Supplementary Information:**

The online version contains supplementary material available at 10.1186/s13046-025-03361-3.

## Introduction

Glioblastoma (GBM), recognized as the foremost frequent and virulent primary brain neoplasm in humans, has a profoundly grim prognosis, with a median survival time of approximately 12–16 months despite exhaustive treatments, which include surgical resection, radiotherapy, and adjuvant chemotherapy [1, 2]. A substantial cause of the notorious therapeutic resistance of GBM is its considerable intratumoral heterogeneity [3]. GBM stem cells (GSCs), a particularly aggressive cell subpopulation within GBM tumors, are distinguished by their self-renewal capability, sustained proliferation, and tumor initiation ability [4, 5]. GSCs have been identified as key promoters of therapeutic resistance, angiogenesis, and immune evasion, thereby forming the basis of the malignant and recurrent nature of GBM [6]. At present, no targeted therapy has effectively addressed GSCs in GBM. Therefore, pinpointing crucial regulators specific to GSCs could shed light on new target therapy for GBM patients.

Metabolic reprogramming, a key hallmark of cancer, plays a critical role in tumor malignancy and resistance to conventional therapies such as radiotherapy and chemotherapy [7, 8]. This phenomenon, in which specific metabolic pathways are abnormally activated in cancer stem cells (CSCs), includes well-known alterations, such as aerobic glycolysis (the Warburg effect), and recent research foci, such as the serine synthesis pathway (SSP) [9, 10]. SSP activity, particularly upregulated in cancers such as breast cancer and melanoma, is a marker of a more aggressive tumor subtype with poorer prognoses [11, 12]. Beyond classical metabolic functions, cancer cells adapt their metabolism to meet increased energy and biosynthesis demands during proliferation [13, 14]. The distinctive metabolic pathways active in tumor cells present novel therapeutic targets, opening new avenues for metabolism-based therapeutic strategies [15].

Phosphoglycerate dehydrogenase (PHGDH), a pivotal rate-limiting enzyme in the SSP, has been linked to the development, progression, and treatment resistance of various tumors [16–18]. However, its function in gliomas, particularly concerning tumor malignancy and radioresistance, has been less explored. In gliomas, PHGDH expression levels positively correlate with WHO grade, and inhibiting PHGDH significantly decreases GBM cell proliferation and invasiveness [19]. Additionally, PHGDH is involved in shaping the tumor microenvironment by orchestrating endothelial cell metabolism within GBM [20]. Despite these findings, the precise role of PHGDH in CSCs of GBM remains unclear.

In this study, we conducted both metabolomic and transcriptomic analyses of human GSCs, revealing that PHGDH plays a crucial role in the pathological regulation of metabolic reprogramming, redox homeostasis, and the DNA damage response in GBM. Targeting PHGDH in GSCs through either genetic ablation or pharmacological inhibition markedly impedes GBM growth and diminishes radioresistance.

## Materials and methods

### Human GBM specimens

The human GBM specimens used in this study were sourced from patients with informed consent at Tongji Hospital, Tongji Medical College of Huazhong University of Science and Technology. These fresh GBM tissues were immediately frozen in liquid nitrogen and stored at − 80 ℃ or fixed with formalin, and embedded in paraffin for immunohistochemical and immunofluorescence staining. All procedures performed in this study were approved by the ethics committee of Tongji Hospital, Tongji Medical College of Huazhong University of Science and Technology (TJ-IRB20220325) and were performed in accordance with the ethical standards of the 2008 Declaration of Helsinki. Detailed information of the patients whose samples were included in the glioma tissue microarray is provided in Supplementary Table 1.

### Cells and cell culture

GSCs were generously provided by professor Jianghong Man (State Key Laboratory of Proteomics, National Center of Biomedical Analysis, China), and obtained from primary GBM samples or patient-derived GBM xenografts, with subsequent functional characterization as previously described [21]. Detailed information of 4121, 387, and 3691 GSCs was provided in our previous study [21]. GSCs were cultured in Neurobasal-A medium (Gibco) with B27 supplement (Gibco), 10 ng/ml EGF (Gold Biotech), 10 ng/ml bFGF (R&D), 1 mM sodium pyruvate (Gibco), and 2 mM L-glutamine (Gibco). GSCs were cultured and differentiated into GDC in Dulbecco’s Modified Eagle Medium (DMEM) containing high glucose (4.5 g/L), supplemented with 1% L-glutamine (200 mM), 1% penicillin/streptomycin, and 10% heat-inactivated fetal bovine serum. Complete media (CM) was formulated to closely match the ingredients of Neurobasal-A medium (0.4 mM serine and 0.4 mM glycine). For serine & glycine deprivation experiments, cells were fed the same media formulation without serine and glycine (-SG media). For serine & glycine rescue experiments, cells were fed the same media formulation with adding serine and glycine (+ SG media, 0.8 mM serine and 0.8 mM glycine).

Specifically, GSC19 was from a GBM patient (48-year old, female); GSC29 from an Astrocytoma patient (Grade III, 53-year old, male); GBM#1 from a GBM patient (44-year old, female); GBM#2 from a GBM patient (56-year old, female); GBM#3 from a GBM patient (60-year old, male). GBM#4 was from a GBM patient (66-year old, male);

Three fetal brain-derived human neural progenitor cell (NPC) lines, namely hNP1, 15,167, and 17,231 (supplied by Lonza), were cultured and maintained in the aforementioned GBM stem cell medium, adhering strictly to the vendor’s guidelines. Meanwhile, NHA cells, procured from Beina Chuanglian Biotechnology Institute (BNCC341796), were sustained in DMEM culture medium enriched with 5 mM glucose and 10% FBS.

### Plasmid construction and lentiviral transfection

To knockdown PHGDH or MYC in GSCs, lentiviral constructs expressing two nonoverlapping short hairpin RNAs (shRNAs) targeting human PHGDH (targeted sequences: #1 CAGGACTGTGAAGGCCTTATT and #2 ACGCTAAGCTGCTGGTGAAAG) and MYC (targeted sequences: #1 CCCAAGGTAGTTATCCTTAAA and #2 ACTGAAAGATTTAGCCATAAT) and nontargeting control shRNA were generated in the pLKO.1 TRC vector (Addgene). To overexpress PHGDH or MYC in GSCs, a lentiviral construct expressing PHGDH or MYC was synthesized by inserting the full-length complementary cDNA of human PHGDH or human MYC into the pCDH-MCS-T2A-Puro-MSCV vector (System Biosciences). All the constructs were transfected into HEK293T cells along with the packaging plasmid pSPAX2 and the envelope plasmid pVSVG (Addgene) in the Neurobasal-A medium with a calcium phosphate transfection kit. After 3 days, lentiviral particles were produced. GSCs were transduced using these lentiviral particles, prior to selection with puromycin (2 μg/ml). The transduction efficiency of each PHGDH construct was confirmed by qPCR and western blotting.

### CRISPR–Cas9 gene knockout

PHGDH single-guide RNAs (sgRNAs) were designed via the CRISPR design tool developed by Broad Institute (https://portals.broadinstitute.org/gpp/public/analysis-tools/sgrna-design). Oligonucleotides were annealed and subsequently inserted into the Lenti-Guide-puro plasmid (Addgene). For the knockout experiments, GSCs that stably expressed Cas9 were generated via transduction with the LentiCas9-blast lentiviral construct (Addgene) and selected using blasticidin (10 μg/ml). Subsequently, these GSC-Cas9 cells were transduced with lentiviral constructs containing the PHGDH sgRNAs, and selection was then carried out using puromycin (2 μg/ml). The knockdown efficiency of the PHGDH sgRNAs was confirmed by western blotting.

### Inducible knockdown

For the inducible knockdown experiments conducted in vitro, lentiviral constructs that carried doxycycline (DOX)-inducible shRNAs were generated in the Tet-pLKO-puro vector (Addgene). GSCs harboring these DOX-inducible PHGDH shRNAs were then cultured in the presence of 100 ng/ml DOX for 4 days. After culture, the cell lysates were harvested to evaluate the knockdown efficiency of the PHGDH shRNAs via quantitative PCR (qPCR) and western blotting analysis. For the in vivo inducible knockdown studies, GSCs harboring the DOX-inducible PHGDH shRNAs were intracranially implanted into nude mice (female, aged 4 weeks). The mice were provided water containing 2 mg/ml DOX. The progression of orthotopic GBM tumors in these models was tracked using bioluminescence imaging, with the Caliper IVIS® Spectrum system from PerkinElmer.

### Cell viability and neurosphere formation assays

For the cell viability assay, we seeded 2,000 cells into each well of a 96-well plate. Viability was assessed on designated days using a CCK8 kit following the manufacturer’s protocol. Additionally, cell proliferation was evaluated using either 5-ethynyl-2’-deoxyuridine (EdU) imaging kits or 5-bromo-2-deoxyuridine (BrdU) incorporation assays in accordance with the instructions provided by the manufacturer. These experiments were conducted in at least triplicate to ensure data reliability. For the neurosphere formation assay, 1,000 GSCs were seeded into each well of a 96-well plate. The number of neurospheres formed was quantified on the sixth day following cell seeding.

### In vitro limiting dilution assays and cell differentiation

GSCs were seeded in individual wells of 96-well plates at the indicated densities (25, 50, 100, 200, or 400 cells per well), with 12 replicates established for each cell density. After ten days, the number of neurospheres emerging in each well was carefully counted. The analysis of these data was conducted using specialized software accessible at http://bioinf.wehi.edu.au/software/elda/.

### Apoptosis assay

The apoptosis assay was performed using an Annexin V-FITC/PI apoptosis kit (Yeasen Biotech, Shanghai) according to the manufacturer’s protocol, and apoptosis was detected by flow cytometry (BD Aria III). For each sample, a total of 10,000 cells were examined, and the subsequent data analysis was carried out utilizing the FlowJo 7.6 software.

### Intracellular ROS quantification

The intracellular level of ROS in GSCs was measured using a Reactive Oxygen Species Assay Kit (H2-DCFDA, Yeasen Biotech, Shanghai, 50101ES) according to the manufacturer’s protocol. Briefly, GSCs were incubated with ROS detection reagent for 30 min, after which the fluorescence intensity (ex = 488/em = 525 nm) was measured via flow cytometry (BD Aria III). Across all samples, a cumulative count of 10,000 cells was scrutinized, with FlowJo 7.6 software facilitating the comprehensive data analysis.

### Intracranial mouse xenografts and combination treatment

All animal experiments performed in this study were approved by the Institutional Review Board of Tongji Hospital, Tongji Medical College of Huazhong University of Science and Technology (protocol code TJH-202206015). Orthotopic GBM xenograft models were established by intracranially transplanting GSCs as previously described [21]. Briefly, GSCs expressing DOX-inducible PHGDH shRNAs (5 × 10^4^ cells) were implanted into the right frontal lobe of nude mice (4 weeks old, female). After indicated days, the tumor-bearing mice were randomized into the vehicle control, DOX (2 mg/ml, daily through the drinking water), irradiation (3 Gy, once per week, 3 times in total), or combination treatment with DOX and irradiation groups. GBM xenograft progression was tracked using bioluminescence imaging with the Caliper IVIS® Spectrum system (PerkinElmer) at designated intervals following tumor implantation. For in vivo serine and glycine deprivation experiment, the mice are either fed with the control diet or diet deprived of serine & glycine [22]. All mice were sacrificed by carbon dioxide inhalation followed by cervical dislocation. Xenograft tissues were removed, snap frozen in liquid nitrogen, stored at − 80 ℃ or preserved in 4% paraformaldehyde (PFA, Sigma-Aldrich) and subsequently embedded in paraffin wax for the purpose of immunohistochemical and immunofluorescence staining procedures.

### PDX models

For PDX models, patient-derived GBM cells (5 × 106 in 100 µL PBS) are subcutaneously implanted into the right armpit of NOD/SCID mice (4 weeks, female). Following the formation of masses, the mice were randomly allocated into four distinct groups: vehicle control, NCT-503 (10 mg/kg, i.p., every 3 days), radiation (3 Gy, once a week, four times in total), or the combination treatment of NCT-503 and IR group. The subcutaneous tumors were measured every 5 days with vernier calipers and the approximate size of the tumor mass was determined utilizing the formula [(minor axis)^2 × (major axis) × 0.5]. The mice were euthanized when the tumor volume surpassed 1500 mm^3^.

### Irradiation

The cells and mice underwent exposure to X-rays emitted by a biological irradiator (RS2000, Rad Source Technologies, Boca Raton, FL). All irradiations were conducted at ambient temperature, maintaining a dosage rate of 100 cGy per minute.

### RNA isolation and real-time PCR

RNA was extracted from GSC or DGCs employing TRIzol reagent (Invitrogen) and subsequently converted into cDNA using a dedicated cDNA synthesis kit (Yeasen Biotech, Shanghai). For quantitative PCR, cDNA was combined with Hieff® qPCR SYBR Green Master Mix (Low Rox Plus) (Yeasen Biotech, Shanghai), and expression was measured with a QuantStudio 1 Real-Time PCR System (Thermo Fisher Scientific). The expression of each target gene’s mRNA was determined comparatively, employing the 2^(-ΔΔCT) technique and adjusting it relative to a stable housekeeping gene (such as GAPDH or β-Actin). The sequences of the primers are provided in Supplementary Table 2.

### Antibodies and reagents

Primary antibodies against PHGDH (14719-1-AP), Caspase 3/p17/p19 (19677-1-AP), PARP1 (13371-1-AP) β-Tubulin (10094-1-AP), β-Actin (66009-1-Ig), GAPDH (60004-1-Ig) were obtained from Proteintech; the anti-phospho-Histone H2A.X (Ser139) antibody (05–636-25UG) was obtained from Millipore; the anti-53BP1 (p53-binding protein 1) antibody (A3859) was obtained from ABclonal Technology; the anti-Olig2 antibody (AF2418), the anti-SOX2 antibody (AF2018), the anti-MYC antibody (AF3696) were obtained from R&D; and the anti-8-OHdG (8-hydroxy-2'-deoxyguanosine, namely 15A3, ab62623) and the anti-GFAP (ab53554) antibodies were acquired from Abcam (Cambridge, UK). Detailed information regarding both the primary and secondary antibodies is provided in Supplementary Table 3. Doxycycline hydrochloride (HY-N0565A), blasticidin A (HY-113542), puromycin dihydrochloride (HY-B1743A), and NCT-503 (HY-101966) were purchased from MedChemExpress (MCE).

### Protein extraction and immunoblot analysis

Total protein was isolated using RIPA buffer (G2002, Servicebio) that was fortified with a phosphatase inhibitor (G2007, Servicebio) as well as protease inhibitors (G2008, Servicebio). To ascertain the protein concentration in the sample, a BCA assay kit was employed. Subsequently, 20–50 µg of protein was loaded and fractionated via sodium dodecyl sulfate–polyacrylamide gel electrophoresis with varying concentrations of 6%, 8%, or 12%. Following this, the proteins were transferred to polyvinylidene fluoride membranes (IPVH00010, Millipore). The membranes were then blocked with NcmBlot blocking buffer (P30500, NCM Biotech) and subjected to incubation with primary antibodies specific to PHGDH (1:3000; Proteintech), SOX2 (1:1000; R&D), Olig2 (1:1000; R&D), MYC (1:1000; R&D), GFAP (1:2000; Abcam), Caspase 3 (1:2000; Proteintech), PARP (1:2000; Proteintech), and γ-H2AX (1:1000; Millipore) at 4 ℃ overnight. After five washes with TBS-T (containing 0.1% Tween), the membranes were incubated with horseradish peroxidase (HRP)-labeled secondary antibodies for 2 h at ambient temperature. Then, a sensitive chemiluminescence reagent (NCM Biotech, Suzhou) was applied to detect and visualize the protein bands.

### Chromatin immunoprecipitation (ChIP)

The ChIP assay was performed using the EZ-Magna ChIP A/G Chromatin Immunoprecipitation Kit (17–10086, Millipore). First, GSCs were subjected to crosslinking with 1% formaldehyde, and crosslinking was terminated by the addition of glycine. Subsequently, chromatin was digested with micrococcal nuclease. To facilitate the breakdown of the nuclear membrane, the lysate was sonicated with multiple pulses. The processed chromatin was then incubated with an antibody specific for MYC (AF3696, R&D) or goat IgG which served as a control. Protein G agarose beads were used to isolate the complexes. The elution process was followed by a reversal of crosslinking by incubation with Proteinase K for 2 h at a temperature of 65 °C. The DNA extracted via this procedure was further analyzed by qPCR. Detailed information on the ChIP-qPCR primer sequences is listed in Supplementary Table 2.

### H&E staining

Hematoxylin–eosin (H&E) staining was utilized to delineate the cancerous lesions in the brains of the different mouse models. Briefly, mice were anesthetized and subjected to cardiac perfusion. Whole brain tissues were gently removed, fixed in 4% formalin, embedded in paraffin, and sliced into sections with a thickness of 7 µm, which were subsequently stained with H&E. Imaging was conducted on a NanoZoomer S360 digital slide scanner (Hamamatsu Japan), and images were analyzed using ZEN Blue software (Zeiss).

### Immunohistochemical and immunofluorescence staining

Immunohistochemical (IHC) and immunofluorescence (IF) staining were performed on human GBM tissues and GBM xenografts as described in our previous publication [23]. The primary antibodies used were as follows: anti-PHGDH (1:100, Proteintech), anti-SOX2 (1:100), anti-Olig2 (1:300, R&D), anti-MYC (1:200, R&D), anti-Caspase 3 (1:200, Proteintech), anti-PARP (1:200, Proteintech), anti-γ-H2AX (1:50, Millipore), anti-53BP1 (1:100, Abclonal), Anti-8-OHdG (15A3) (1:100, Abcam). Briefly, GBM tissues were gathered, stabilized in 4% paraformaldehyde, encased in paraffin, and sliced into 7 μm sections. The prepared slides were then dewaxed, rehydrated, and underwent antigen retrieval following established protocols. Subsequently, the slides were blocked with a PBS mixture containing 1% bovine serum albumin (BSA) and 0.3% Triton X-100 for a duration of 2 h at room temperature. Following incubation with the specified primary and secondary antibodies, the slides were counterstained with DAPI and subsequently mounted on glass with antifade mountant (G1401, Servicebio) for IF or dehydrated and mounted with mounting medium (WG10004160, Servicebio) for IHC and were then evaluated by microscopy (CKX53, Olympus, Japan).

### Luciferase assay

The PHGDH-luc vector (Promega) and the internal control vector pRL-TK (Promega) were co-transfected into GSCs utilizing Lipofectamine 3000 (Invitrogen). Following a 48-h incubation, a luciferase reporter assay was performed as outlined by the manufacturer’s guidelines (Promega). The resulting luciferase activity data was then calibrated to the values obtained from the pRL-TK vector to ensure accurate analysis.

### Metabolomic analysis and metabolite profiling

Metabolites were extracted from 1 × 10^7^ GSCs in 250 ml of 50:30:20 (methanol: acetonitrile: 10 mM Tris, pH 9.3) extraction buffer. The extraction samples were subsequently centrifuged for 5 min at 20,000 × g, after which the supernatants were transferred to liquid chromatography-tandem mass spectrometry (LC–MS) vials. Nontargeted LC–MS/MS analysis was professionally performed by BIOTREE in Shanghai, China. Briefly, an ultra-high-performance liquid chromatography (UHPLC) system (Agilent Technologies 1290, Santa Clara, CA) equipped with a UPLC BEH Amide column (1.7 μm 2.1*100 mm, Waters Corporation, Milford, MA) was used. This system was connected to a TripleTOF 6600 mass spectrometer (Q-TOF, AB Sciex, Redwood City, CA), which was used to acquire MS/MS spectra on an information-dependent basis (IDA) during LC/MS experiments. For data handling, raw MS data files were converted to mzXML format using ProteoWizard software. The R package XCMS (version 3.2) was then used for further data processing. This preprocessing procedure yielded a comprehensive data matrix encompassing retention time (RT) values, mass-to-charge ratio (m/z) values, and peak intensities. For peak annotation post-XCMS processing, the R package CAMERA was utilized. Metabolite identification was performed with the MS2 database. The metabolomic data were analyzed using the MetaboAnalystR R package. To identify significantly altered metabolites, a 2-tailed Mann–Whitney U test was conducted. Metabolites with FDR (adjusted *P* values) of less than 0.05 were considered to be significantly differentially abundant.

### RNA sequencing

Samples of transfected GSCs were collected and processed using TRIzol for RNA extraction, followed by sequencing at Beijing Genomics Institute (BGI; Shenzhen, China). The sequencing data were initially processed using SOAPnuke (v1.5.2, BGI) for quality filtering. The resulting clean reads were converted to FASTQ format and subsequently aligned to the reference genome using HISAT2 (v2.0.4; Hopkins, Baltimore, MD, USA). Analysis of fusion genes and differentially spliced genes (DSGs) was carried out using Ericscript (v0.5.5) and rMATS (V3.2.5; Sourceforge, San Diego, CA, USA), respectively. Further alignment of the clean reads to both known and novel transcripts was performed using Bowtie2 (v2.2.5; Hopkins, Baltimore, MD, USA), with a comprehensive database developed by BGI that includes coding transcripts. Gene expression levels were quantified using RSEM (v1.2.12). Differential gene expression was analyzed using DESeq2 (v1.4.5; UNC, Chapel Hill, NC, USA), setting a Q value threshold of ≤ 0.05. Functional enrichment analyses, including Gene Ontology (GO) and Kyoto Encyclopedia of Genes and Genomes (KEGG) enrichment analyses, were also conducted using Phyper based on the hypergeometric test, with the Bonferroni correction applied to adjust the significance levels for term and pathway enrichment, while maintaining a stringent threshold (Q value ≤ 0.05).

### LC–MS analysis of NCT-503

Homogenized mouse brain tissues and extracted serum samples were initially treated with a prechilled (− 80 °C) 1:1 mixture of 80% methanol and acetonitrile and were then incubated at − 80 °C for an hour. They were then centrifuged at 18,000 × g for 10 min, and the supernatant was carefully desiccated in a SpeedVac system. The dried residues were redissolved in 100 µl of a 50% methanol solution, followed by a further centrifugation step to acquire the supernatants. Finally, the prepared samples and standards underwent analysis with a Triple Quad 6500 mass spectrometer (SCIEX), coupled to an UltiMate 3000 HPLC system (Thermo Fisher Scientific), as previously described [21]. The LC separation was efficiently carried out utilizing an ACQUITY UPLC HSS T3 column (100 mm × 2.1 mm, Waters). To analyze NCT-503 through the technique of multiple reaction monitoring (MRM), the detection was set at the 409/169.4 transition in the positive ion mode. Peak integration and subsequent statistical analysis were conducted using MultiQuant™ 2.1 software, also from SCIEX.

### Statistics and reproducibility

Statistical analysis was conducted with GraphPad Prism 9.5.1 software. The data are reported in the form of Mean ± SEM or Mean ± SD and represent findings from a minimum of three separate experimental runs. For comparing two groups, a Student’s t-test was employed, whereas for comparisons involving three or more groups, a one-way ANOVA test was utilized. The Kaplan–Meier method and log-rank test were adopted to assess survival rates. A *p*-value below 0.05 was deemed statistically significant. The IC50 value of NCT-503 was determined through nonlinear regression analyses based on dose–response curves. The present study involved examining the relationship between gene expression levels by analyzing various datasets from The Cancer Genome Atlas (TCGA), the Chinese Glioma Genome Atlas (CGGA), and the NCBI Gene Expression Omnibus (GEO) database. Gene set variation analysis (GSVA) was used to score pathway enrichment. The stemness score was calculated using the “TCGAanalyze_Stemness” package [24].

## Results

### PHGDH is strongly upregulated in GSCs

**Fig. 1:**
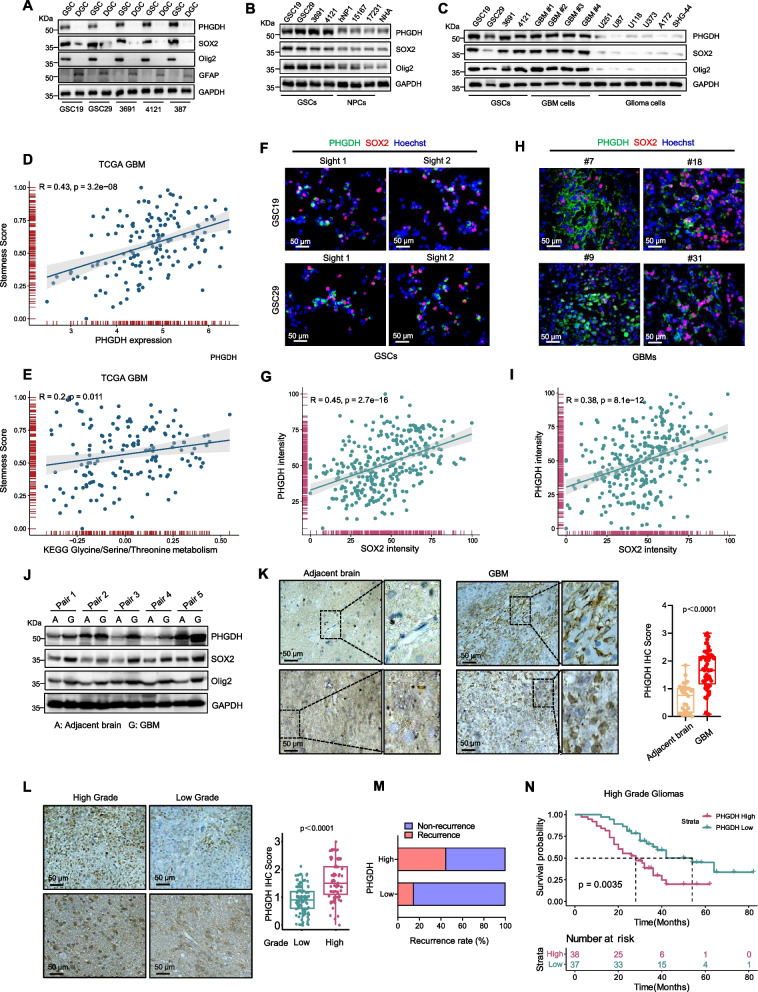
PHGDH is strongly upregulated in GSCs. **A**-**C** Immunoblot (IB) displaying the levels of the specified proteins in glioma stem-like cells (GSCs) compared to the differentiated GBM cells (DGC) (**A**), in GSCs relative to human neural progenitor cells (hNP1, 15167, and 17231) and normal human astrocyte (NHA) (**B**), and in GSCs in contrast to primary GBM cells and traditional glioma cell lines (**C**). **D** and **E** Dot plots showing the results of correlation analyses between the tumor stemness score computed via the TCGAbiolinks algorithm and the PHGDH transcription level (**D**), and between the tumor stemness score and the Glycine/Serine/Threonine metabolic pathway score determined through GSVA (**E**) in the TCGA-GBM cohort. **F** and **G** Representative immunofluorescence (IF) images of the GSC19 and GSC29 cell lines showing staining for PHGDH (green) and SOX2 (red). Nuclei are highlighted by Hoechst counterstain (blue) (**F**). The relationship between the PHGDH and SOX2 staining intensities in the GSC lines, as determined by the Pearson correlation coefficient, is depicted (**G**). **H** and **I** Illustrative IF images of primary human glioblastoma (GBM) tissues displaying staining for PHGDH (green) and SOX2 (red). Nuclei are highlighted using Hoechst counterstain (blue) (**H**). The scale bars represent 50 μm. The graph shows the quantified intensity of PHGDH staining in SOX2 positive (*n* = 300) compared with SOX2 negative (*n* = 300) cells in five randomly selected fields across four tumor specimens. The Pearson correlation coefficient reflecting the association between the PHGDH and SOX2 staining intensities in GBM cells is also presented (**I**). **J** IB analysis revealed the expression levels of PHGDH, SOX2, and Olig2 in human primary GBM tissue samples compared to the adjacent normal brains. **K** The immunohistochemical (IHC) image highlighting PHGDH staining in primary GBM tissues compared with the corresponding adjacent brain tissues. The scale bars represent 50 μm. A graph detailing the PHGDH IHC scores for the respective groups is also shown (**K**, right). **L**-**N** IHC staining was used to assess PHGDH expression in a glioma tissue microarray. Included are representative images and boxplots detailing PHGDH histoscores for both low-grade and high-grade gliomas (**L**). The scale bars represent 50 μm. (low-grade, *n* = 100; high-grade, *n* = 75). The recurrence percentages of the gliomas are displayed: those with low PHGDH expression (*n* = 13) versus those with high expression (*n* = 39) (**M**). Kaplan–Meier survival curves depicting the survival outcomes of patients with either low PHGDH expression (*n* = 37) or high PHGDH expression in high-grade glioma (*n* = 38) (**N**). The analysis was performed with the log-rank (Mantel-Cox) test. Supplementary Table 1 provides additional details. The employed statistical tests included unpaired two-sided Student’s t-test (**G**, **I**, **K**) and Welch’s two-sided t-test (**L**)

To verify the increasing expression of PHGDH in GSCs, we performed immunoblot (IB) and qRT-PCR analyses of several GSCs and differentiated GBM cells (DGCs), which were derived from GBM patients or patient-derived xenografts (PDXs), as previously described [21, 25–27]. GSCs and DGCs were characterized by the expression of stem cell markers (Olig2, Sox2 and Nestin) or the differentiation marker GFAP (Fig. 1A and Supplementary Figure S1A) and were further functionally validated by using in vivo limiting dilution assays and serial transplantation assays respectively (Supplementary Figure S1B-D). We found PHGDH expression was markedly higher than that in the corresponding DGCs (Fig. 1A and Supplementary Figure S2A). Additionally, PHGDH levels decreased as GSCs underwent serum-induced differentiation (Supplementary Figure S2B). PHGDH expression was also higher in GSCs and tumor cells derived from GBM samples compared to neural progenitor cells (NPCs), normal human astrocytes (NHAs), and conventional glioma cell lines (Fig. 1B, C).

In addition, we investigated the relationships among the tumor stemness score determined with the TCGAbiolinks algorithm [24], the PHGDH transcription level, and the serine metabolic pathway score determined through GSVA in the TCGA-GBM cohort. Our analysis highlighted a significant positive correlation between the tumor stemness score and both the PHGDH mRNA level (*R* = 0.43) and the enrichment score of the serine metabolic pathway (*R* = 0.2) (Fig. 1D, E). In the CGGA database, we also noted a consistent positive association between the tumor stemness score and the PHGDH expression level, regardless of tumor grade (Supplementary Figure S2C, D). Additionally, co-immunofluorescence (co-IF) staining indicated that cells with high PHGDH levels also expressed SOX2 or Olig2 in both GSC lines and primary GBM samples (Fig. 1F, H and Supplementary Figure S2E, G). Notably, Pearson’s correlation analysis revealed a strong positive correlation between the PHGDH expression level and the expression levels of SOX2 and Olig2 in both GSC lines and GBM tissues (Fig. 1G, I and Supplementary Figure S2F, H).

Clinically, IB analysis of fresh GBM specimens showed elevated PHGDH expression compared to normal brain tissues (Fig. 1J). IHC staining revealed strong PHGDH expression in GBM cells but minimal detection in normal brain tissues (Fig. 1K and Supplementary Figure S2I). Furthermore, IHC analysis of a glioma tissue microarray indicated that higher PHGDH levels in high-grade gliomas compared to low-grade ones, and patients with high PHGDH IHC scores were more prone to recurrence and had lower survival rates (Fig. 1L-N, Supplementary Figure S2J and Supplementary Table 1). Collectively, these findings highlight the elevated expression of PHGDH in GSCs and its association with advanced progression in glioma.

### PHGDH amplifies GSC self-renewal and accelerates tumor progression

**Fig. 2:**
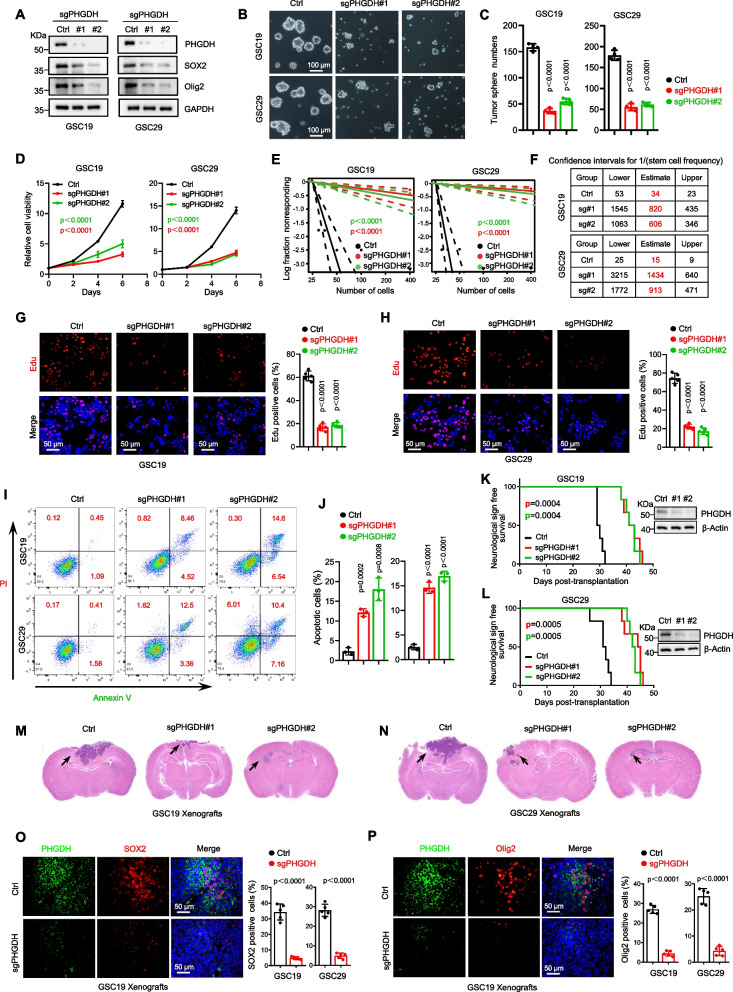
PHGDH amplifies GSC self-renewal and accelerates tumor progression. **A** Using the CRISPR-Cas9 technique, we modified GSCs by PHGDH knockout (KO). IB analysis shows the levels of the proteins of interest in both control and PHGDH KO GSCs. **B** Photographs of tumorspheres (initiated from 2,000 cells per well) derived from either control or PHGDH KO GSCs are presented. The scale bar represents 100 μm. **C** A statistical summary of the number of tumorspheres is provided (mean ± SD, derived from four independent biological experiments). **D** GSC proliferation was notably curtailed upon PHGDH KO, as revealed by cell viability assays. (The Data are represented as the means ± SD of three independent biological replicates). **E** In vitro ELDAs revealed a decreased propensity for tumorsphere formation in PHGDH KO GSCs. **F** The tables present the estimated stem cell frequencies in both the control and PHGDH-KO groups. **G** Immunofluorescence imaging of EdU incorporation in GSC19 after PHGDH KO (left), with a subsequent statistical breakdown of EdU-positive cells (analyzing > 1,000 cells for each group, (mean ± SD, images *n* = 5, from 5 biologically independent samples). The reference scale bar represents 50 μm. **H** Similarly, EdU incorporation in GSC29 following PHGDH KO is illustrated (left), accompanied by a numerical representation of the EdU-positive cells (after observing > 1,000 cells from each category, mean ± SD, images *n* = 5, from 5 biologically independent samples) (right). The scale bar represents 50 μm. **I** The apoptotic tendencies of control and PHGDH KO GSCs were evaluated using flow cytometry, from three independent biological replicates. **J** Quantification of apoptotic cells among control and PHGDH KO GSCs (expressed as the means ± SD derived from three independent biological replicates). **K**, **L** Control and PHGDH KO GSCs (5 × 10.^4^ cells/mouse) were transplanted into the brains of nude mice (nu/nu, *n* = 6). Kaplan–Meier survival analysis of these mice was performed using the log-rank (Mantel-Cox) test, and the results are shown. A representative IB is shown, and the data highlight the PHGDH knockout efficiency in the xenograft samples (upper section). **M**, **N** Representative images of H&E-stained sections of mouse brains collected 30 days post-GSC transplantation are shown. The reference scale bar represents 1 mm. **O** Combined immunofluorescence (co-IF) staining was used to evaluate PHGDH (green) and SOX2 (red) expression in GBM xenografts generated from either control or PHGDH KO GSC19. On the right, quantification of SOX2 positive cells is shown, with the data presented as the means ± SD, based on five images obtained from five distinct biological samples. Cell nuclei were contrasted using Hoechst staining (blue). The corresponding scale bar represents 50 μm. **P** In a similar manner, co-IF staining revealed the presence of PHGDH (green) and Olig2 (red) within GBM xenografts derived from control or PHGDH KO GSC19. The graph on the right shows the data for Olig2 positive cells, shown as the mean ± SD, based on images from five different fields from five separate biological samples. Statistical analysis was performed using one-way ANOVA (**D**-**E**), unpaired two-sided Student’s t-test (**C**, **G**, **H**, **J**), and Welch’s two-sided t-test (**O**, **P**)

To investigate the functional impact of PHGDH in GSCs, we employed the CRISPR-Cas9 technique for genetic knockout (KO) of PHGDH in two GSC lines (GSC19 and GSC29). Effective suppression of endogenous PHGDH expression was achieved using two distinct sgRNAs, which also considerably reduced the SOX2 and Olig2 protein levels in GSCs (Fig. 2A). These observations were corroborated by PHGDH knockdown (KD) in GSCs using DOX-induced shRNAs (Supplementary Figure S3A). Remarkably, either KO or inducible-KD of PHGDH markedly inhibited the self-renewal ability of GSCs, as evidenced by the result of the tumor sphere formation experiments (Fig. 2B, C and Supplementary Figure S3B), the diminished GSC viability (Fig. 2D and Supplementary Figure S3C), and by the results of the in vitro limiting dilution assay (Fig. 2E, F). Additionally, PHGDH KO in GSCs hampered cell proliferation, as indicated by EdU incorporation (Fig. 2G, H). Inducible-KD of PHGDH hindered GSC expansion, as determined by BrdU uptake (Supplementary Figure S3D, E). Both KO and KD PHGDH induced apoptosis, as determined by flow cytometric analysis (Fig. 2I, J and Supplementary Figure S3F, G).

To determine the influence of PHGDH on GSC-mediated tumor initiation, we generated orthotopic GBM xenografts by implanting either control or PHGDH-depleted GSCs into the brains of immunodeficient mice. PHGDH depletion extended symptom-free survival (Fig. 2K, L) and resulted in smaller tumors (Fig. 2M, N). In vivo PHGDH expression was manipulated by injecting mice with GSCs expressing luciferase and DOX-induced PHGDH shRNA (Luc/DOX-shPHGDH), and DOX treatment upon tumor establishment drastically inhibited tumor growth (Supplementary Figure S3H, J) and extended lifespan (Supplementary Figure S3I, K). PHGDH ablation decreased SOX2 and Olig2 positive tumor cells in GBM xenografts (Fig. 2O, P; Supplementary Figure S3L, M), indicating a reduced GSC reservoir. Overall, these findings demonstrate that PHGDH ablation impairs GSC proliferation and tumor progression.

### PHGDH specifically maintains redox homeostasis, one-carbon metabolism, and DNA repair through the serine synthesis pathway

**Fig. 3:**
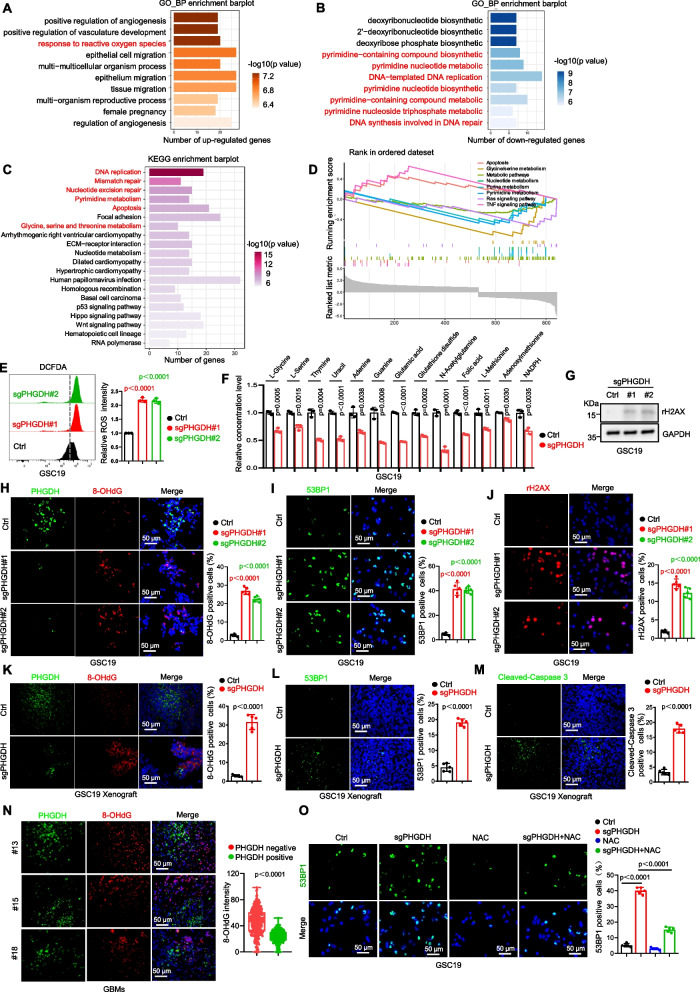
PHGDH specifically maintains redox homeostasis, one-carbon metabolism, and DNA repair through serine synthesis pathway. **A**-**D** Transcriptome profiling via RNA sequencing was conducted in control and PHGDH knockout (KO) GSC19. Gene Ontology (GO) term enrichment analysis based on the RNA-seq data revealed enrichment (**A**) and depletion (**B**) of distinct gene sets in PHGDH KO GSCs relative to controls. Additionally, Kyoto Encyclopedia of Genes and Genomes (KEGG) pathway enrichment analysis was performed with the DEGs (**C**), complemented by Gene Set Enrichment Analysis (GSEA) (**D**). **E** Peroxide levels were quantified by measurement of the H2-DCFDA fluorescence intensity via flow cytometry in PHGDH KO GSCs; relative ROS intensity measurements are displayed (mean ± SD, *n* = 3 independent biological replicates). **F** Non-targeted metabolomic profiling was performed to compare control and PHGDH KO GSC19. **G** IB analysis showed differential γH2AX protein levels between control and PHGDH-KO GSC19. **H** Co-IF staining for PHGDH (green) and 8-OHdG (red) in control and PHGDH KO GSC19, with quantifications of 8-OHdG positive cells (mean ± SD, images *n* = 5, from 5 biologically independent samples). **I** IF staining for 53BP1 (green) was conducted in control and PHGDH KO GSCs, and 53BP1 positive cells were quantified (mean ± SD, images *n* = 5, from 5 biologically independent samples). **J** IF staining for γH2AX (red) in control and PHGDH KO GSCs, with quantification of γH2AX positive cells (mean ± SD, images *n* = 5, from 5 biologically independent samples). **K** Co-IF staining for PHGDH (green) and 8-OHdG (red) in GSC19-derived xenografts, with quantification of 8-OHdG positive cells (mean ± SD, images *n* = 5, from 5 biologically independent samples). **L** and **M** IF staining for 53BP1 (green, **L**) and cleaved-Caspase 3 (green, **M**) in GSC19-derived xenografts, including quantification of positive staining cells (mean ± SD, images *n* = 5, from 5 biologically independent samples). **N** Co-IF staining for PHGDH (green) and 8-OHdG (red) in primary GBM specimens, with quantification of the 8-OHdG staining intensity in PHGDH negative (*n* = 240) and PHGDH positive (*n* = 300) cells (right). **O** IF staining for 53BP1 (green) in control and PHGDH-KO GSCs treated with either vehicle control or NAC (5 mM), along with quantification of 53BP1 positive cells (mean ± SD, *n* = 5 independent biological replicates). Nuclei were counterstained with Hoechst in all IF images (blue). The scale bars represent 50 μm. Statistical analysis was conducted using Welch’s two-sided t-test (**E**, **G**, **N**, **O**), and unpaired two-sided Student’s t-test (**H**-**M**) where appropriate

RNA-sequencing analysis revealed that PHGDH absence was found to modify (predominantly increase) the expression levels of a considerable number of genes in GSCs (Supplementary Figure S4A). GO analysis indicated that PHGDH KO upregulates genes related to the cellular response to ROS (Fig. 3A), while down-regulated genes involve pyrimidine metabolism and DNA replication/repair (Fig. 3B). KEGG analysis of differentially expressed genes showed significant enrichment in DNA replication/repair, pyrimidine metabolism, apoptosis, and serine metabolism pathways in PHGDH KO GSCs (Fig. 3C), confirmed by GSEA analysis (Fig. 3D). Furthermore, we found that DOX stimulation did not affect the cell viability of GSCs (Supplementary Figure S4B).

Redox status is pivotal for the maintenance of CSCs and their resistance to therapy. To validate the influence of PHGDH deletion on ROS levels, we performed staining with the redox-sensitive probe H2-DCFDA in GSCs. Flow cytometric analysis revealed that PHGDH KO notably augmented peroxide production (Fig. 3E), and this finding was further substantiated in GSCs with inducible-KD of PHGDH (Supplementary Figure S4C). As PHGDH is a pivotal metabolic enzyme in the SSP, we further employed targeted metabolomic analyses to elucidate how PHGDH influences the metabolism of GSCs. After PHGDH deletion in GSCs, levels of one-carbon metabolism metabolites decreased to varying degrees, such as L-glycine, L-serine, thymine, uracil, folic acid, L-methionine, adenosylmethionine, and NADPH (Fig. 3F and Supplementary Figure S4D). Increased γH2AX levels, indicating DNA double-strand break responses [28], were observed post-PHGDH deletion (Fig. 3G and Supplementary Figure S4E), supporting RNA-seq enrichment analysis results.

Moreover, we conducted co-IF staining for PHGDH and 8-OHdG (a DNA oxidative damage marker) in GSCs cultured in vitro. GSCs with PHGDH depletion exhibited significantly elevated levels of 8-OHdG, indicating that PHGDH may play a role in mitigating ROS-induced DNA damage (Fig. 3H and Supplementary Figure S4F). Consistent with the role of PHGDH in DNA repair, the expression of 53BP1, a critical mediator in the repair of DNA double-strand breaks, was also markedly increased in PHGDH-depleted GSCs via IF staining (Fig. 3I and Supplementary Figure S4G). In agreement with the IB results, enhanced γH2AX immunoreactivity was observed in GSCs lacking PHGDH (Fig. 3J and Supplementary Figure S4H). In vivo co-IF staining for PHGDH and 8-OHdG in GSC-derived xenografts and human GBM specimens revealed lower 8-OHdG levels in high PHGDH-expressing tumor cells, indicating PHGDH’s protective effect against oxidative DNA damage (Fig. 3K, N, Supplementary Figure S4I). Increased 53BP1 and cleaved-Caspase 3 protein levels upon PHGDH deletion in GSC xenografts (Fig. 3L, M, Supplementary Figure S4J, K) suggest compromised DNA repair mechanisms, leading to increased DNA damage and apoptosis in GSCs.

To elucidate the role of ROS in GSC maintenance and DNA damage post-PHGDH loss, GSCs with PHGDH KO or inducible-KD were exposed to N-acetyl-L-cysteine (NAC), an antioxidant. NAC treatment neutralized the peroxide surge, restored stem cell marker expression, reduced 53BP1 immunoreactivity, and reversed γH2AX level increases caused by PHGDH deletion (Fig. 3O, Supplementary Figure S4L-Q). These findings indicate that PHGDH-induced DNA damage is partially mediated by ROS. Overall, PHGDH maintains redox homeostasis, one-carbon metabolism, and DNA repair via the SSP.

Cells can either uptake exogenous serine or synthesize it from the glycolytic intermediate 3-phosphoglycerate (3-PG) via the serine biosynthesis pathway [29]. To determine whether serine deprivation mimics the biological effects of PHGDH deletion, we assessed the proliferation of GSCs cultured in complete medium (CM) and medium deficient in both serine and glycine (-SG). We found that the viability of GSC19 cells was significantly reduced under serine and glycine deprivation (Supplementary Figure S5A). Additionally, serine deprivation led to increased peroxide production (Supplementary Figure S5B) and a decrease in key one-carbon metabolites in GSC19 cells (Supplementary Figure S5C). IF staining revealed significantly elevated levels of 8-OHdG and 53BP1 in GSC19 cells cultured in -SG medium (Supplementary Figure S5D-E), a trend that was also observed in GSC19 xenografts (Supplementary Figure S5F-G). Notably, serine deprivation prolonged symptom-free survival in GSC19 xenografts (Supplementary Figure S5H). Conversely, adding serine and glycine (+ SG) can rescue the effects of PHGDH deletion on increased peroxide production, elevated 53BP1 levels, and the decrease in key one-carbon metabolites in GSC19 cells (Supplementary Figure S5I-K). Together, these findings suggest that serine deprivation induces effects comparable to PHGDH deletion in GSCs.

### MYC drives the expression of PHGDH in GSCs

**Fig. 4:**
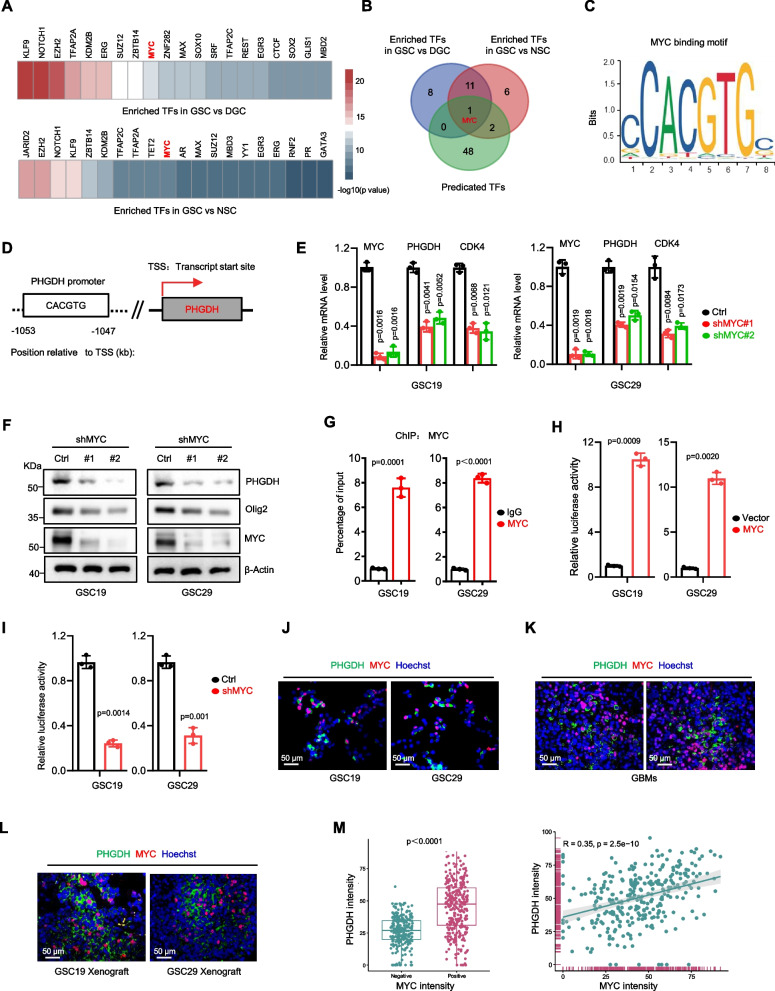
Myc drives the expression of PHGDH in GSCs. **A** Heatmap depicting the 20 most significantly enriched transcription factors identified as potentially responsible for DEGs between GSCs and DGCs/NSCs, based on the GSE54791 and GSE119834 datasets. **B** Venn diagram illustrating the overlap between transcription factors with differential expression profiles in GSCs compared with DGCs/NSCs and those predicted to regulate PHGDH. **C** Schematic depiction of the MYC-binding motif. **D** The promoter of PHGDH harbors a conserved MYC-binding element. **E** Quantitative RT-PCR analysis was conducted to measure the mRNA levels of MYC, PHGDH, and CDK4 following targeted MYC knockdown via two distinct shRNAs in GSC19 and GSC29. The data are presented as the mean ± SD values and were derived from three separate biological replicates to ensure experimental validity. **F** IB analysis was utilized to quantify the protein levels of MYC, PHGDH, and Olig2 after MYC knockdown using two unique shRNAs in the aforementioned GSC lines. **G** Chromatin immunoprecipitation (ChIP) assays were carried out on the PHGDH promoter utilizing the designated antibodies. The resulting immunoprecipitates were analyzed by qPCR with primers flanking the PHGDH promoter region to determine the binding affinity of MYC at this site. **H**, **I** The luciferase activity of the PHGDH promoter-driven reporter construct was quantified post-cotransfection with a MYC overexpression plasmid (**H**) or shMYC (**I**) in the GSC lines GSC19 and GSC29, revealing the transcriptional activation by MYC. The data are presented as the mean ± SD of three biologically independent experiments. **J**-**L** Co-IF staining showing the spatial co-localization of PHGDH (green) and MYC (red) in GSCs in culture (**J**), GSC-derived xenografts in vivo (**L**), and in human GBM tissue samples (**K**), providing visual evidence of their co-expression. Nuclei are counterstained with Hoechst in all IF images (blue). The scale bars represent 50 μm. **M** A quantitative evaluation of MYC-positive cells within human GBM specimens was performed, providing a statistical representation of MYC expression levels across different samples. Statistical analyses were performed using Welch’s two-sided t-test (**E**, **N**) and unpaired two-sided Student’s t-test (**G**-**I**) where appropriate

To elucidate the upstream molecular mechanisms driving high PHGDH expression in GSCs, we explored the transcriptional regulation of the PHGDH promoter. Utilizing a computational biology framework called Lisa, we identified the top 20 enriched transcription factors responsible for differentially expressed genes (DEGs) between GSCs and DGCs/ neural stem cells (NSCs), which included 12 common transcription factors (Fig. 4A, B) [30]. Employing bioinformatics prediction methods, we found that among the top-ranking transcription factors for PHGDH, MYC was the sole candidate overlapping with the differentially expressed transcription factors associated with GSCs (Fig. 4B). Analysis of the PHGDH nucleotide base sequence revealed the presence of a conserved MYC-binding element (CACGTG) in its promoter region (Fig. 4C, D), suggesting MYC’s regulatory role in PHGDH expression, supported by previous studies [31, 32]. MYC knockdown led to a reduction in both the mRNA and protein levels of PHGDH in GSCs (Fig. 4E and F) in analysis with CDK4, a recognized MYC target, serving as the positive control [33]. Conversely, MYC overexpression elicited increases in PHGDH levels in GSCs (Supplementary Figure S6A, B). ChIP-qPCR with a MYC-specific antibody confirmed significant MYC occupancy on the PHGDH promoter (Fig. 4G). Moreover, PHGDH-luciferase reporter assay demonstrated that MYC overexpression enhanced PHGDH-luc activity, while MYC knockdown reduced it (Fig. 4H, I). Additionally, co-IF staining showed high MYC expression correlated with elevated PHGDH levels in GSCs, GSC-derived xenografts, and human GBM specimens (Fig. 4J-M).

MYC suppression increased peroxide production in GSCs (Supplementary Figure S6C) and upregulated DNA damage markers 8-OHdG and 53BP1 (Supplementary Figure S6D, E). Mice with shMYC GSC-derived xenografts had extended symptom-free survival and smaller tumors compared to controls (Supplementary Figure S6F, G). Targeted metabolomics revealed MYC knockdown reduced metabolites such as L-glycine, L-serine, thymine, adenine, and folic acid in GSCs (Supplementary Figure S6H). Increased 8-OHdG and 53BP1 levels upon MYC deletion were also observed in the GSC xenograft model (Supplementary Figure S6I, J). Rescue experiments showed that PHGDH overexpression in shMYC GSCs reversed the effects of MYC knockdown on PHGDH expression, oxidative stress, and DNA damage (Supplementary Figure S6K-M). These results establish that MYC regulates PHGDH transcription in GSCs.

### PHGDH promotes GSC radioresistance through redox homeostasis and DNA repair

**Fig. 5:**
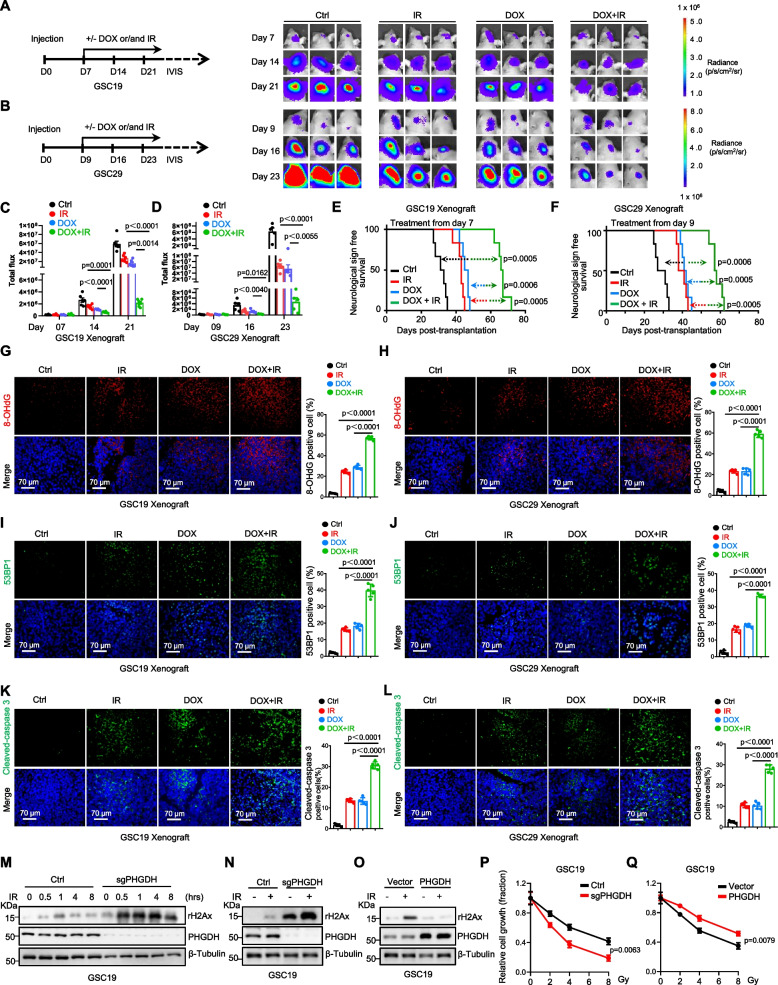
PHGDH promotes radioresistance in GSCs through redox homeostasis and DNA repair. **A**-**F** Nude mice (nu/nu) were surgically engrafted intracranially with GSCs labeled with luciferase and transduced with a DOX-inducible system for shPHGDH expression. Mice harboring either GSC19 (**A**, **C**, **E**) or GSC29 (**B**, **D**, **F**) were randomly assigned to treatment groups (6 mice per group). Starting from day 7 or day 9 post-engraftment, as per the study design (**A**, **B**, left), treatments were administered: control, 3 Gy ionizing radiation (IR) delivered weekly over three sessions, 2 mg/ml DOX in the drinking water or a combination of IR and DOX. The progression of GBM xenografts was monitored through bioluminescence imaging, and representative images are displayed (**A**, **B**, right). The quantified bioluminescence signals, reflecting tumor growth, are plotted (**C**, **D**, mean ± SEM, analyzed using an unpaired two-sided Student’s t-test). The survival of the mice is depicted in Kaplan–Meier plots (**E**, **F**) and was analyzed with the log-rank (Mantel-Cox) test. **G**-**L** IF staining for the DNA damage and apoptosis markers 8-OHdG (red, **G**, **H**), 53BP1 (green, **I**, **J**), and cleaved-caspase 3 (green, **K**, **L**), was performed on GBM xenograft sections. Representative images are presented (left), and the quantification of cells positive for these markers is graphically represented (right) (mean ± SD, *n* = 5, biologically independent samples, analyzed using an unpaired two-sided Student’s t-test). The scale bars in all the images represent 70 μm. **M**-**O** IB analysis was used to measure the level of γH2AX in control and PHGDH KO GSCs with IR exposure for different time points (**M**), in control and PHGDH KO GSCs with or without 1-h IR of exposure (**N**), and in GSCs overexpressing PHGDH and treated with or without IR for 1 h (**O**). IR, 3 Gy. **P**-**Q** Cell viability assays were conducted on control and PHGDH KO GSCs subjected to various doses of IR for 1 h (**P**) and on GSCs overexpressing PHGDH and treated with different IR doses for 1 h (**Q**). The viability data are presented as the means ± SEMs and were analyzed by two-way ANOVA for statistical comparison

IR induces DNA damage directly through double-strand breaks and indirectly by boosting ROS levels [34, 35]. DNA repair mechanisms, dependent on nucleotide synthesis, are tightly regulated by the interplay between amino acid and one-carbon metabolic pathways, wherein PHGDH is an essential participant in this intricate biochemical network. To explore the potential of ROS augmentation and impairment of nucleotide synthesis via PHGDH suppression to enhance the sensitivity of GBM to radiotherapy, we developed orthotopic GBM xenograft models with GSCs engineered to express Luc/DOX-shPHGDH. After the tumors had grown to comparable sizes, the mice were randomized and subjected to various treatments: control, IR alone (3 Gy, weekly for three weeks), DOX (administered daily in drinking water), or a combination of IR and DOX (Fig. 5A, B). The findings revealed that suppression of PHGDH robustly inhibited tumor proliferation in the GBM models (Fig. 5A-D) and notably increased survival rates (Fig. 5E, F). While IR, as a standalone therapy, effectively inhibited tumor expansion and marginally increased survival (Fig. 5A-F), ablation of PHGDH markedly boosted tumor susceptibility to IR, leading to the most substantial increase in survival across all treatment modalities (Fig. 5A-F). Further analysis via 8-OHdG and 53BP1 staining indicated that both PHGDH suppression and IR independently increased DNA damage, but their combination drastically intensified this effect (Fig. 5G-J). Additionally, cleaved-caspase 3 staining showed increased apoptosis with each treatment alone, but significantly higher cell death with the combined treatment (Fig. 5K, L), supporting the hypothesis that PHGDH depletion sensitizes GBM to IR therapy.

Moreover, in vitro studies revealed that IR treatment escalated peroxide production in GSCs, further amplified by PHGDH knockout (Supplementary Figure S7A, J). Ablation of PHGDH significantly enhanced IR-induced apoptosis in GSCs, as demonstrated by the increased levels of cleaved-caspase 3 and cleaved-PARP observed via IB analysis (Supplementary Figure S7C, L) and by the augmented Annexin V and PI staining detected via flow cytometry (Supplementary Figure S7E, G, N, P). In contrast, PHGDH overexpression mitigated the peroxide accumulation triggered by IR in GSCs (Supplementary Figure S7B, K) and markedly reduced apoptosis in GSCs post-IR treatment (Supplementary Figure S7D, F, H, M, O, Q).

IR induced DNA damage in GSCs, indicated by the elevated expression of the markers 53BP1 and γH2AX, as assessed by both IB analysis and IF staining (Fig. 5M, N and Supplementary Figure S7I, R). Notably, PHGDH knockout alone increased the levels of these DNA damage markers, indicating that the loss of PHGDH independently induces DNA damage, even without IR exposure (Fig. 5M, N and Supplementary Figure S7I, R). Furthermore, the absence of PHGDH impaired the restoration process of DNA damage caused by IR in GSCs (Fig. 5M, N and Supplementary Figure S7I, R), underlying the heightened sensitivity of GSCs to radiotherapy. Interestingly, the adverse effects of DNA damage inflicted by the combination of PHGDH knockout and IR were counteracted by reintroducing PHGDH in vitro (Fig. 5O). This finding highlights the notable synergy between PHGDH knockout and IR in terms of cell lethality, which is reversible through PHGDH reintroduction (Fig. 5P, Q).

Collectively, these findings suggest that elevated PHGDH levels afford GSCs a protective advantage against IR-induced cell death by diminishing ROS buildup and repairing DNA damage. Conversely, targeting PHGDH could act in concert with radiation therapy to enhance its therapeutic efficacy against GBM, offering a compelling avenue for improving treatment outcomes.

### Treatment with the inhibitor NCT-503 mimics the effects of PHGDH deletion

**Fig. 6:**
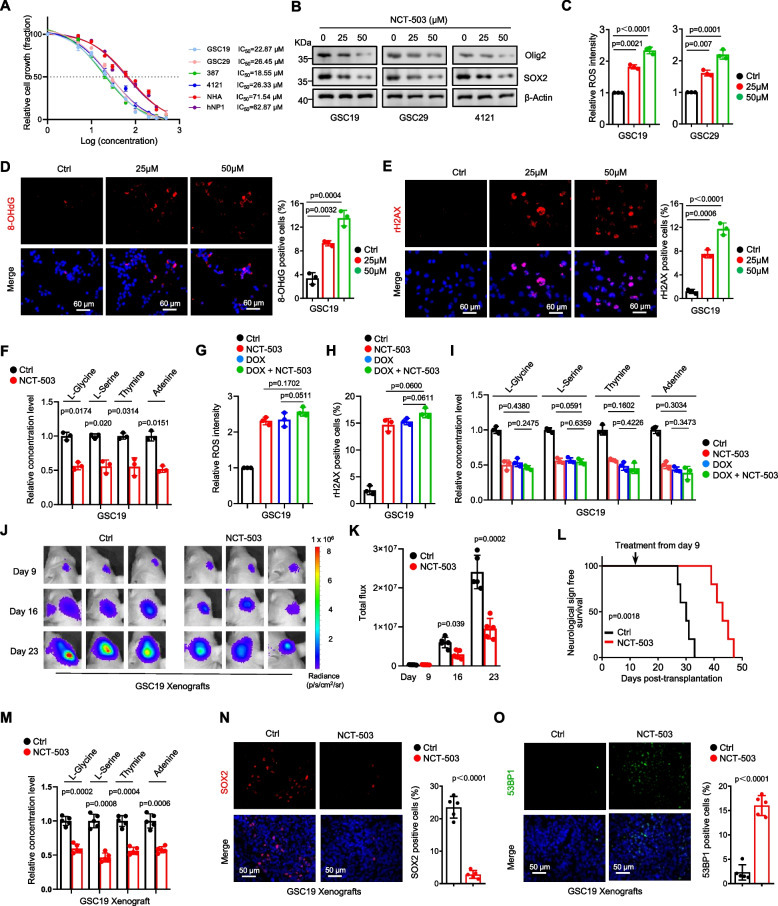
Treatment with the inhibitor NCT-503 mimics the effects of PHGDH deletion. **A** Dose–response curves were generated to ascertain the sensitivity of multiple GSC lines, along with NHA and hNP1 cells, to NCT-503. After a 48-h treatment period with increasing doses of NCT-503, the IC50 values were deduced through nonlinear regression analysis using dose–response data (mean ± SD, *n* = 3, biologically independent experiments). **B** IB was used to measure protein expression levels in GSCs following a 12-h treatment with varying concentrations of NCT-503. **C** Peroxide production, as indicated by H2-DCFDA fluorescence, was quantified via flow cytometry in GSC19 treated with incremental concentrations of NCT-503 for 18 h (mean ± SD, *n* = 3, biologically independent experiments). **D**-**E** IF staining was performed to visualize 8-OHdG (red, **D**) and γH2AX (red, **E**) in GSC19 GSCs treated with graded concentrations of NCT-503. Images showing the staining are presented on the left, and the quantification of positively stained cells is shown on the right (mean ± SD, *n* = 3, biologically independent samples). A scale bar representing 70 μm is included for reference. **F** A targeted metabolomics approach was used to detect metabolic alterations between control and NCT-503 treated GSC19 cells. **G** Peroxide levels in control GSC19 and inducible PHGDH-knockdown treated with NCT-503 were measured via flow cytometry. **H** Quantitative analysis of IF staining for γH2AX in control or PHGDH inducible-knockdown GSC19 after NCT-503 treatment was carried out (mean ± SD, *n* = 3, biologically independent samples). **I** Comparative targeted metabolomic analyses were conducted to elucidate metabolic differences between control GSC19 and inducible PHGDH-knockdown upon NCT-503 treatment. **J**-**K** Nude mice intracranially implanted with luciferase-labeled GSC19 were separated into groups (*n* = 6) on Day 9 post-implantation and treated with NCT-503 (10 mg/kg/day) or left untreated. The progression of GBM xenografts was monitored through bioluminescence imaging, with representative images (**J**) and quantification of bioluminescence signals indicating tumor growth (**K**) displayed. **L** A Kaplan–Meier survival curve was constructed for the treated mice and the data were analyzed using the log-rank (Mantel-Cox) test. **M** A targeted metabolomics approach was used to detect metabolic alterations between control and NCT-503 treated GSC19 xenografts. **N** IF staining was conducted to detect SOX2 in GBM xenografts subjected to various treatments, with representative images shown on the left and quantification of SOX2-positive cells on the right. **O** Similarly, IF staining for 53BP1 was performed on GBM xenografts subjected to various treatments, with visual representations on the left and counts of 53BP1-positive cells on the right. Nuclei were counterstained with Hoechst (blue). Scale bars, 70 μm. Statistical analyses were performed using Welch’s two-sided t-test (**C**-**I**, **K**) and unpaired two-sided Student’s t-test (**M**-**O**)

NCT-503, a potent inhibitor of the enzyme PHGDH involved in serine synthesis within the glycolytic pathway, has been demonstrated to disrupt this metabolic process [36]. We examined the impact of NCT-503 on the survival of GSCs and observed dose-dependent suppression of cellular proliferation across various GSC lines (Supplementary Figure S8A). Measurement of the half-maximal inhibitory concentration (IC50) revealed that even at low concentrations (< 30 μM), NCT-503 markedly inhibited of GSC proliferation, compared with NPC and NHA (Fig. 6A). Following treatment with NCT-503, there was a reduction in the expression of stem cell markers, which exhibiting dose dependency (Fig. 6B and Supplementary Figure S8B). Additionally, NCT-503 significantly promoted peroxide generation in GSCs, and this effect intensified with increasing concentration (Fig. 6C). Concurrently, dose-dependent increases in the DNA damage markers 8-OHdG and γH2AX were detected in GSCs after NCT-503 treatment (Fig. 6D and E). Targeted metabolomic analysis further revealed that NCT-503 considerably depleted key cellular metabolites, such as L-glycine, L-serine, thymine, and adenine, in GSCs (Fig. 6F).

To examine whether the effects of NCT-503 on GSCs are exerted via suppression of PHGDH activities, we administered NCT-503 to both control GSCs and GSCs with inducible knockdown of PHGDH with NCT-503. Interestingly, the increase in the peroxide level (Fig. 6G, left), increase in the γH2AX level (Fig. 6H, left), and decreases in metabolite concentrations (Fig. 6I, left) were revealed in NCT-503 treatment. However, these trends were less pronounced in PHGDH-depleted GSCs than in control cells (Fig. 6G-I). These findings suggested that NCT-503 exerts its effects specifically through targeting PHGDH.

To evaluate the potential of NCT-503 as a viable therapeutic agent for GBM in vivo, we administered NCT-503 intraperitoneally to both normal mice and those implanted with orthotopic GBM xenografts and then measured its bioavailability within the brain. Mass spectrometry analyses indicated a significant accumulation of NCT-503 within the brain tissue, with notably greater concentrations detected within the tumor-bearing brain regions than within normal brain tissue (Supplementary Figure S8C, D). These findings suggested that NCT-503 is capable of effectively crossing the blood–brain barrier and concentrating within brain tumors to potentially exert its antitumor effects. Crucially, treatment with NCT-503 substantially inhibited tumor progression (Fig. 6J, K) and significantly extended the lifespan (Fig. 6L). Additionally, levels of key one-carbon metabolites, including L-glycine, L-serine, thymine, and adenine, were substantially reduced in GSC-derived xenografts following NCT-503 treatment (Fig. 6M). These findings align with previous observations from PHGDH-knockdown studies in GSC-derived xenografts. Moreover, NCT-503 treatment led to a decrease in the number of tumor cells expressing the stem cell markers SOX2 and Olig2 (Fig. 6N and Supplementary Figure S8E), an increase in the expression of markers associated with DNA repair (Fig. 6O and Supplementary Figure S8F), and an increase in tumor cell apoptosis (Supplementary Figure S8G). Notably, NCT-503 did not exhibit overt toxicity in vivo; moreover, it did not result in weight reduction among the mice (Supplementary Figure S8H), nor did it induce any morphological changes in the brain, liver, lung, or kidney, as evaluated by H&E staining (Supplementary Figure S8K-N). Additionally, it did not affect the survival of NPCs within the subventricular zone (SVZ) of the brain (Supplementary Figure S8I and J). Conversely, adding serine and glycine (+ SG) can rescue the effects of NCT-503 on increased peroxide production, elevated 53BP1 levels in GSC19 cells (Supplementary Figure S8O-P). Taken together, these findings indicate that NCT-503 preferentially targets GSCs and impedes the growth of orthotopic GBM xenografts while sparing normal tissues from harmful side effects.

### Pharmacological targeting of PHGDH increases the sensitivity of GSCs to IR

**Fig. 7:**
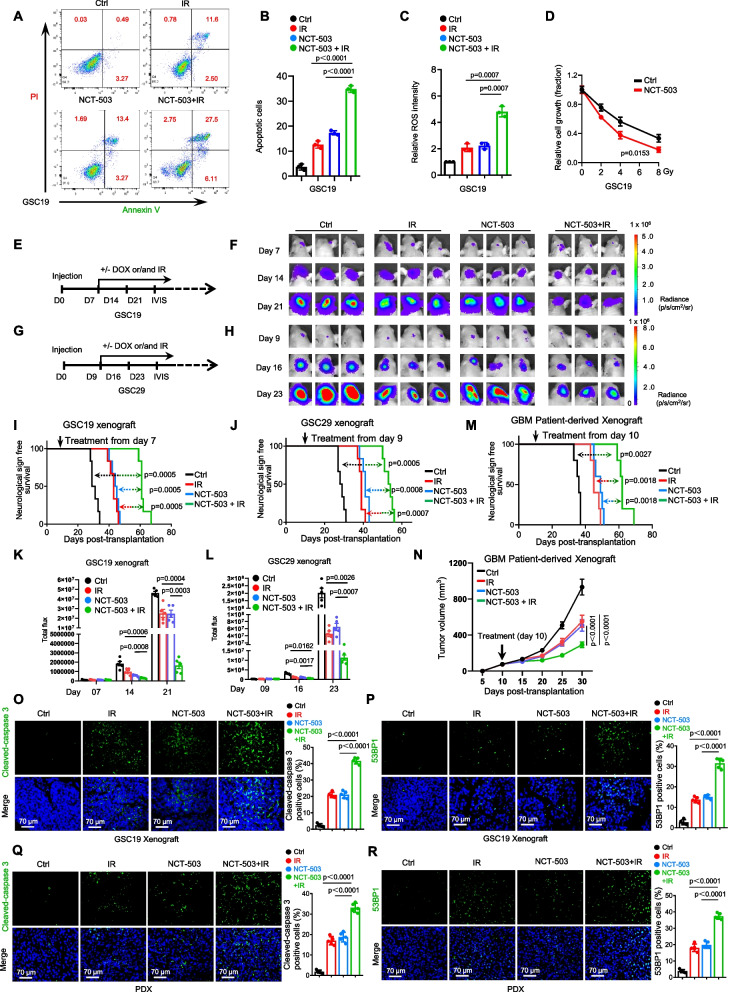
Pharmacological targeting of PHGDH increases the sensitivity of GSCs to IR. **A** Apoptosis in GSC19 was quantified using flow cytometry after the indicated treatments for 48 h, with IR administered at a dose of 3 Gy. **B** Quantitation of apoptotic cells is shown. **C** Peroxide levels in the GSC19, as indicated by H2-DCFDA fluorescence, were also measured using flow cytometry after treatment for the same duration and with the same IR dosage. **D** The growth kinetics of GSC19 exposed to varying doses of IR for 48 h, with and without the addition of NCT-503 (30 μM), were assessed, and the data were normalized to the baseline growth of untreated cells in each experimental cohort. **E**-**L** For in vivo analyses, nude mice (nu/nu) intracranially implanted with GSCs expressing luciferase were divided into random treatment groups (**E**-**F**, GSC19, *n* = 6 mice per group; **G**-**H**, GSC29, *n* = 6 mice per group) and administered either a control treatment, IR alone (3 Gy, once weekly for three sessions), NCT-503 alone (10 mg/kg/day), or a combination of IR and NCT-503 starting from either day 7 (GSC19 xenografts) or day 9 (GSC29 xenografts). The progression of GBM xenografts was monitored via bioluminescence imaging with representative images (**F** and **H**) and quantitative analyses of bioluminescence intensities representing tumor growth (**K** and **L**) presented. Kaplan–Meier survival curves for the mice are shown (**I**, GSC19 xenograft; **J**, GSC29 xenograft) (log-rank (Mantel-Cox) test). **M**-**N** Further, NOD/SCID mice subcutaneously implanted with GBM patient-derived xenograft (PDX) tumors were assigned to treatment groups (*n* = 5 mice per group) on day 11 to receive the control treatment, IR (3 Gy, once weekly for four sessions), NCT-503 (10 mg/kg/day), or the combination therapy. Survival outcomes are shown on a Kaplan–Meier curve (**M**), while tumor volumes were measured and recorded (**M**). **O**-**P** Representative images of immunofluorescence (IF) staining for cleaved-caspase 3 (**O**) and 53BP1 (**P**) in GSC-derived xenografts are shown. Quantitative data for cells with positive staining are also reported. **Q**-**R** Representative images of IF staining for cleaved-caspase 3 (**Q**) and 53BP1 (**R**) in GBM PDX tumors are shown, with corresponding quantitative analyses (right). Cell nuclei were counterstained with Hoechst (blue). The scale bars represent 70 μm. Statistical analyses were performed using two-way ANOVA (D, N), and Welch’s two-sided t-test (**B**, **C**, **J**, **L**, **O**-**R**)

We then investigated whether the PHGDH inhibitor NCT-503 can enhance the effects of IR in the treatment of GBM. Our findings revealed that the combined application of NCT-503 and IR substantially increased apoptosis in GSCs (Fig. 7A, B) and elevated peroxide production (Fig. 7C). Moreover, treating GSCs with NCT-503 increased their sensitivity to IR in vitro (Fig. 7D). Crucially, in two orthotopic GBM xenograft models, the combination regimen of NCT-503 and IR was the most effective at suppressing tumor proliferation and prolonging survival, with more potent effects than either NCT-503 or IR treatment alone (Fig. 7E-L). The superiority of this combined therapeutic approach was further corroborated in GBM patient-derived xenografts (PDXs) (Fig. 7M, N). Concurrently, staining for cleaved-caspase 3 in GSC or PDX Xenografts revealed a substantial increase in tumor cell apoptosis upon combination treatment (Fig. 7O, Q). Moreover, staining for 53BP1 demonstrated notable augmentation of DNA damage repair mechanisms, corroborating the potent synergism of NCT-503 and ionizing radiation (IR) in inhibiting the progression of GBM (Fig. 7P, R). Taken together, these data suggest that targeting PHGDH with NCT-503 effectively diminishes the growth of tumors originating from GSCs and markedly bolsters the therapeutic impact of radiotherapy in GBM management.

## Discussion

Aberrant activation of specific metabolic pathways in CSCs, termed metabolic reprogramming, is a crucial event involved in tumor malignancy and resistance to radiotherapy and chemotherapy [30, 37]. Intervening in these CSC-specific metabolic processes and redox homeostasis to selectively eradicate CSCs is a current focus in the field of oncological therapy development [38]. Our study revealed that PHGDH expression is elevated in GSCs and GBM, contributes to the maintenance of tumorigenic stem-like features, and is associated with a poorer prognosis in GBM patients. Inhibition of PHGDH markedly diminishes the GSC self-renewal capability of GSCs both in vitro and in vivo by modulating redox homeostasis and one-carbon metabolism which subsequently activates DNA repair via the SSP. Genetically or pharmacologically targeting PHGDH enhances GBM cell sensitivity to radiotherapy, indicating the potential of PHGDH as an innovative therapeutic target in GBM management.

Postoperative radiotherapy is established as the standard treatment for GBM [39]. Although radiotherapy can prolong survival in GBM patients, resistance to radiation is a common and substantial challenge, as evidenced by the observation that almost all patients eventually develop resistance to this treatment [40]. A critical factor in this context is the role of oxidative stressors, such as ROS produced by radiotherapy [41]. Compared with bulk tumor cells (BTCs), GSCs have been found to have lower ROS levels; these lower ROS levels are believed to enhance GSC self-renewal and contribute to resistance against antitumor therapies [42]. Furthermore, serine is crucial not only for protein and lipid synthesis but also for one-carbon metabolism (1CM), facilitating nucleotide synthesis [43]. Additionally, 1CM is a source of NADPH production through methylenetetrahydrofolate dehydrogenase (MTHFD) [44], essential for regenerating reduced glutathione and thioredoxin, which combat oxidative stress [45]. Our study, incorporating transcriptomic and metabolomic analyses, revealed that PHGDH disruption leads to increased enrichment of the GO term response to ROS and decreases key metabolites such as folic acid and glutathione. Inhibiting PHGDH specifically increases peroxide production, counteracted by ROS scavenger NAC. IR treatment and PHGDH knockout synergistically increase peroxide production in GSCs, whereas PHGDH overexpression mitigates IR-induced peroxide accumulation. These findings highlight the important role of PHGDH in modulating oxidative stress responses in GSCs, particularly in the context of radiotherapy.

Another mechanism contributing to GBM radioresistance involves DNA damage and repair, which occurs primarily through the activation of DNA damage response (DDR) signaling [4, 46]. IR causes DNA damage directly by inducing double-strand breaks and indirectly by increasing ROS levels [35]. Serine, a key one-carbon source in cancer cells, plays a vital role in the metabolism of amino acids and the synthesis of nucleotides and is essential for cancer cell proliferation and DNA repair mechanisms [47]. By disrupting PHGDH expression to interfere with serine metabolism, we observed that the genes with altered expression were involved primarily in the DNA replication and nucleotide metabolism pathways. Inhibiting PHGDH led to increased production of 8-OHdG, phosphorylation of H2AX, and formation of 53BP1 foci in GSCs. Additionally, combining IR with PHGDH ablation synergistically enhanced DNA damage in GSCs, subsequently triggering cellular apoptosis.

In the TCGA PanCancer Atlas Studies, PHGDH genomic amplification is detected in various cancers, such as melanoma and breast cancer, linked to overexpression at chromosome 1p [12, 48]. However, no reports indicate PHGDH gene amplification in GBM, suggesting alternative mechanisms other than copy number variations may govern PHGDH expression in GBM, potentially involving transcription factor activation. MYC, a key regulator of metabolism, influences multiple metabolic pathways, including glycolysis and glutaminolysis [49]. Crucially, PHGDH, along with PSAT1 and PSPH, are known targets of MYC and are particularly activated under conditions of nutrient deprivation [32]. Additionally, PHGDH has been identified as a direct transcriptional target of ATF4, which was found to be transcriptionally activated by NRF2 in a non-small cell lung cancer model [11]. By integrating the predictions of the top-ranking transcription factors for PHGDH and conducting a comprehensive analysis of the top 20 enriched transcription factors potentially responsible for the differential gene expression between GSCs and DGCs/NSCs, we identified MYC as the principal regulator of PHGDH in GSCs. Functional validation experiments revealed that either upregulation or downregulation of MYC could affect the expression of PHGDH. Furthermore, in GBM tissues and GBM xenografts, the expression of MYC was positively correlated with PHGDH expression. ChIP assays also confirmed the binding of MYC to the promoter region of PHGDH. In alignment with the role of PHGDH in DNA repair, the knockdown of MYC significantly elevated the levels of the DNA damage markers 8-OHdG and 53BP1 in GSCs. Therefore, the increase in PHGDH expression in GBM is at least partially regulated by MYC.

NCT-503 sourced from the NIH Molecular Libraries Small Molecule Repository (MLSMR), was identified as a targeted inhibitor of PHGDH [36]. The pharmacokinetic profile of NCT-503 in the plasma, liver, and brain following a single intraperitoneal administration of 10 mg/kg revealed greater drug enrichment in the brain than in the serum, suggesting efficient penetration of NCT-503 through the blood–brain barrier. Previous studies have shown that NCT-503 enhances the therapeutic efficacy of TMZ in GBM by acting as a chemosensitizer [50]. In our current study, treatment with NCT-503 significantly inhibited tumor growth in GSC models and conferred survival advantages, with no evident indications of illness or morbidity observed in the mice-bearing tumors. Mass spectrometry analyses indicated a substantial accumulation of NCT-503 within the brain, with notably higher concentrations detected in tumor-bearing brain regions than in normal brain tissue. Furthermore, combining NCT-503 with radiotherapy exhibited a potent synergistic effect on tumors in established orthotopic GBM xenografts and PDXs of GBM. These findings validate the feasibility of using NCT-503 for the clinical treatment of GBM, as both a standalone treatment and an adjuvant therapy alongside radiation treatment.

## Conclusions

In summary, our findings indicate that MYC is a key regulator of PHGDH expression in GSCs, and contributes to the maintenance of redox homeostasis, one-carbon metabolism, and the DNA repair response via the SSP. This regulation ultimately leads to the inhibition of mitochondrial ROS accumulation, thereby promoting GSC self-renewal and radioresistance. Our results also demonstrate that targeting GSCs through either genetic deletion or pharmacological inhibition of PHGDH effectively suppresses GBM growth and mitigates resistance to radiation therapy, highlighting that PHGDH blockade may synergize with radiotherapy in the treatment of GBM.

## Supplementary Information

Supplementary Material 1.

## Data Availability

All the data used and analyzed in this study are available within the Article and the Supplementary sources. Images of the original western blotting gels are available in Supplementary material. The transcriptomic data and other additional data will be made available upon request.

## Abbreviations

GSCs: Glioma stem-like cells
GBM: Glioblastoma
PHGDH: Phosphoglycerate dehydrogenase
SSP: Serine synthesis pathway
ROS: Reactive oxygen species
DGCs: Differentiated GBM cells
PDXs: Patient-derived xenografts
NPCs: Neural progenitor cells
NHAs: Normal human astrocytes
SVZ: Subventricular zone
IR: Ionizing radiation
DDR: DNA damage response
