# Supplementary material for: PHGDH activation fuels glioblastoma progression and radioresistance via serine synthesis pathway
Source: J Exp Clin Cancer Res. 2025 Mar 19;44:99. doi: 10.1186/s13046-025-03361-3 (PMC11921657; doi:10.1186/s13046-025-03361-3)
Supplement: Supplementary file 1 — Supplementary Material 1. [file 13046_2025_3361_MOESM1_ESM.pdf]

Supplementary Figure Legends

Figure S1. Characterization and validation of GSC19 and GSC29

A, qRT-PCR analysis of stem-cell markers Olig2, Sox2 and Nestin in 5 different GSC and DGC pairs (GSC19, GSC29, 387, 4121, and 3691).

B-C, in vivo limiting dilution assay of GSCs and DGCs. Kaplan-Meier survival curves of nude mice intracranially implanted with either GSCs or DGCs for lines GSC19 (**B**) and GSC29 (**C**) (n=5-6 mice for each group). The number of transplanted cells is indicated. Significance testing was analyzed by log-rank test.

D, GSCs tumor initiation in the in vivo serial transplantation. 5×10^3 GSCs (GSC19 or GSC29) were intracranially injected into the brains of nude mice (nu/nu) for the primary xenograft. All recipients developed tumor and died within 60 d, and these tumor cells (5×10^3) could be serially transplanted at the second round and the third round, suggesting the long-term self-renewal and tumorigenicity of GSCs. Kaplan–Meier survival curves of mice implanted with GSCs are shown (left). Summary of the serial transplantation experiment is shown (right).

Figure S2. PHGDH is strongly upregulated in GSCs

A, Quantitative RT-PCR was employed to assess the mRNA levels of PHGDH across various GSCs and their corresponding DGCs. Results are represented as mean ± SD from three biologically independent experiments.

B, Using IB, we evaluated the expression levels of PHGDH, SOX2, Olig2, and GFAP during the differentiation of GSCs over specified days. The differentiation process was initiated using 10% FBS.

C and **D,** Dot plots show the correlation analysis between the tumor stemness score derived from the TCGAbiolinks algorithm and PHGDH transcription levels in CGGA-693 GBM cohort (**C**) and CGGA-693 LGG cohort (**D**).

E and **F,** Representative immunofluorescence (IF) images from the GSC19 and GSC29 cell lines show staining of PHGDH (in green) alongside Olig2 (in red). The nuclei are accentuated with Hoechst counterstain (in blue) (**E**). The relationship between PHGDH and Olig2 staining intensities in the GSC lines, as measured by the Pearson correlation coefficient, is depicted (**F**).

G and **H,** Illustrative IF images of primary human GBM samples displaying staining for PHGDH (in green) and Olig2 (in red). Nuclei are highlighted using Hoechst counterstain (in blue) (**G**). Scale bars represent 50 μm. The Pearson correlation coefficient reflecting the association between PHGDH and Olig2 staining intensities in GBM cells is also presented (**H**).

I, IHC analysis of PHGDH in primary GBM samples juxtaposed with their corresponding adjacent brain tissues. The accompanying images use a 50 μm scale bar for reference.

J, Depicted are characteristic images from the IHC staining of PHGDH, capturing recurrent gliomas (samples A9 and B6, both Grade III) in comparison to non-recurrent gliomas (samples H3 and F2, both Grade III) sourced from a glioma tissue microarray. These visions also employ a 50 μm scale bar for context.

The employed statistical tests include unpaired two-sided Student's t-test (**A, F, H**).

Figure S3. PHGDH amplifies GSC self-renewal and accelerates tumor progression

A, GSCs, which were genetically modified for DOX-inducible-shPHGDH expression, were exposed to either DOX (100 ng/ml) or a control substance. IB was utilized to examine the levels of designated proteins.

B, Statistical analysis highlighting the quantity of tumor spheres (initiated with 2000 cells per well) generated by either the control or GSCs with inducible PHGDH knockdown. Data is represented as mean ± SD, stemming from four distinct biological tests.

C, Cell viability assays indicated a suppression in GSC proliferation upon PHGDH inducible-KD. Data is illustrated as Mean ± SD, based on findings from three individual biological studies.

D, Photographic depiction of BrdU assimilation in GSC19 following PHGDH inducible-KD is provided on the left, with a chart on the right showing the percentage of BrdU-positive cells among over 1,000 cells per group. The scale bar is 50 µm.

E, Similarly, BrdU incorporation in GSC29 cells after PHGDH KD is depicted on the left, with a summary of BrdU-positive cell percentages for over 1,000 cells per group on the right. Scale bar: 50 µm.

F, Flow cytometry was employed to quantify apoptotic tendencies in GSCs, comparing the control group to those with PHGDH inducible-KD.

G, Quantification of the apoptotic cells in Ctrl or PHGDH KD GSCs (Mean ± SD, n=3, biologically independent experiments).

H-K GSCs, equipped with DOX-inducible-shPHGDH and a luciferase reporter, were introduced into the brains of nude mice (nu/nu). Starting from day 9 (GSC19) or day 10 (GSC29), mice either received a vehicle control or DOX (2 mg/ml in drinking water) to trigger the expression of shPHGDH. The progression of GBM xenografts was monitored at indicated days via bioluminescence (on the left in **H, J**). A quantified assessment of tumor growth based on bioluminescence is provided (on the right in **H, J**) and is characterized by mean ± SEM values, gathered from five biologically distinct mice. Survival trajectories of the mice, as portrayed by Kaplan– Meier curves, are presented (**I, K**) (GSC19, n = 5; GSC29, n = 5; Log-rank Mantel-Cox test). The effectiveness of the PHGDH suppression in the xenografts is demonstrated alongside through IB.

L and **M,** Co-IF images spotlight the presence of PHGDH (in green) and either SOX2 or Olig2 (in red) within the GBM xenografts originating from either the control or the GSCs with PHGDH inducible knockdown (GSC19). Quantification of SOX2-positive or Olig2-positive cells is respectively showcased on the right. The data is expressed as mean ± SD, based on images from five different fields, each obtained from five separate biological samples. The cell nuclei are distinctly highlighted using a Hoechst stain, which appears blue, with an accompanying scale bar measuring 50µm (**L, M**). Statistical analysis was performed using one-way ANOVA (C), unpaired two-sided Student’s t-test (B, D, E, G, H, J), and Welch’s two-sided t-test (L, M).

Figure S4. PHGDH specifically maintains redox homeostasis, one-carbon metabolism, and DNA repair through the serine synthesis pathway

A, Transcriptomic profiling via RNA sequencing was conducted in control and PHGDH knockout (KO) GSC19. The heatmap depicted the relative expression levels of genes, identifying 311 genes downregulated and 532 genes upregulated in PHGDH KO cells compared to controls ($p < 0.05$, fold change > 2). For visualization, log2-transformed raw data were employed and a color gradient was applied to represent expression levels across the spectrum (**Left**). A Volcano plot provided a visual representation of these relative expression changes, marking the significance versus the magnitude of change (**Right**).

B, Cell viability assays of GSCs upon Dox administration. Data is illustrated as Mean ± SD, based on findings from three individual biological studies.

C, Peroxide levels were quantified using H2-DCFDA fluorescence by flow cytometry in PHGDH KO GSC29, with relative ROS intensity measurements displayed (mean ± SD, n = 3 independent biological replicates).

D, Targeted metabolomic approach was utilized to assess the metabolic distinctions between control and PHGDH KO GSC29.

E, IB analysis demonstrated the differential levels of γH2AX proteins between control and PHGDH KO GSC29.

F, Co-IF staining for the co-localization of PHGDH (green) with 8-OHdG (red) was observed, and quantitative analysis of 8-OHdG positive cells was presented (mean ± SD, images n =5, from 5 biologically independent samples).

G and H, IF staining of 53BP1 (green, **G**) and γH2AX (red, **H**) in both control and PHGDH KO GSCs allowed for the enumeration of positive cells (mean ± SEM, images n =5, from 5 biologically independent samples).

I, Co-IF staining for PHGDH (green) and 8-OHdG (red) in GSC-derived xenografts (GSC29), with quantifications of 8-OHdG positive cells (mean ± SD, images n =5, from 5 biologically independent samples).

J and **J,** IF staining for 53BP1 (green, **J**) and cleaved-Caspase 3 (green, **K**) in GSC29-derived xenografts, including quantifications of positive staining cells (mean ± SD, images n =5, from 5 biologically independent samples).

L, Control or PHGDH KO GSC19 were subjected to treatment with either vehicle control or N-acetylcysteine (NAC, 5 mM) for 36 hours. Flow cytometry analysis of peroxide levels was conducted post-treatment, alongside a quantification of the ROS intensity (mean ± SD, n = 3 independent biological replicates).

M, This process was mirrored in control or DOX-inducible shPHGDH GSC29 treated similarly, where the analysis of peroxide and quantification of ROS intensity post-treatment was depicted (mean ± SD, n = 3 independent biological replicates).

N and **O**, For both control or PHGDH KO GSC19 (**N**) and control or DOX-shPHGDH GSC29 (**O**) treated with vehicle control or NAC, IB was performed to ascertain levels of PHGDH, SOX2, and Olig2.

P, IF staining of 53BP1 (green) in control and PHGDH KO GSC29 treated with vehicle control or NAC (5 mM) was executed, with a quantitative assessment of 53BP1 positive cells (mean ± SD, images n =5, from 5 biologically independent samples).

Q, IB was used to analyze γH2AX levels in control, and PHGDH KO GSCs (GSC19 left, GSC29 right) treated with vehicle control or NAC (5 mM) are shown. Nuclei were counterstained with Hoechst in all IF staining (blue). Scale bars represent 40 μm.

Statistical analyses were performed using Welch's two-sided t-test (C, E, L, M, P) and unpaired two-sided Student's t-test (F-K) where appropriate.

Figure S5. Serine deprivation reveals the same effects to PHGDH deletion

A, Cell viability was evaluated at predetermined time intervals of GSC19 cells grown in complete medium (CM) and medium lacking serine and glycine (-SG) (Mean ± SD, n=3, biologically independent experiments).

B, Peroxide levels were quantified using H2-DCFDA fluorescence by flow cytometry GSC19 cells grown in CM and -SG medium, with relative ROS intensity measurements displayed (mean ± SD, n = 3 independent biological replicates).

C, A targeted metabolomics approach was used to detect metabolic alterations between GSC19 cells cells grown in CM and -SG medium.

D, IF staining for 8-OHdG (red) was conducted in GSC19 cells grown in CM and -SG medium, and 8-OHdG positive cells were quantified (mean ± SD, images n =5, from 5 biologically independent samples).

E, IF staining for 53BP1 (green) was conducted in GSC19 cells grown in CM and -SG medium, and 53BP1 positive cells were quantified (mean ± SD, images n =5, from 5 biologically independent samples).

F-G, IF staining for 8-OHdG (red, **F**) and 53BP1 (green, **G**) in GSC29-derived xenografts in both control and dietary serine and glycine starvation, including quantifications of positive staining cells (mean ± SD, images n =5, from 5 biologically independent samples).

H, Survival trajectories of the mice of GSC19-derived xenografts in both control and dietary serine and glycine starvation as portrayed by Kaplan–Meier curves, are presented (n = 5; Log-rank Mantel-Cox test).

I, Control or PHGDH KO GSC19 were subjected to treatment with either CM or medium adding serine and glycine (+SG). Flow cytometry analysis of peroxide levels was conducted post-treatment, alongside a quantification of the ROS intensity (mean ± SD, n = 3 independent biological replicates).

J, IF staining of 53BP1 in control and PHGDH KO GSC19 treated with either CM or medium adding serine and glycine (+SG), with a quantitative assessment of 53BP1 positive cells (mean ± SD, images n =5, from 5 biologically independent samples).

K, A targeted metabolomics approach was used to detect metabolic alterations between control and PHGDH KO GSC19 treated with either CM or medium +SG.

Figure S6. MYC drives the expression of PHGDH in GSCs

A, Quantitative RT-PCR analyses were conducted to evaluate the mRNA expression levels of Myc and PHGDH after MYC overexpression in GSC19 and GSC29.

B, IB was utilized to determine the protein expression levels of MYC and PHGDH following the forced overexpression of MYC in GSC19 and GSC29.(mean ± SD, n = 3 independent biological replicates).

C, Following targeted knockdown of MYC with two specific shRNAs in GSC19 and GSC29, peroxide production was assessed using the H2-DCFDA assay and quantified by flow cytometry. ROS intensity measurements are displayed (mean ± SD, n = 3 independent biological replicates).

D and **E,** IF staining for 8-OHdG (red, **D**) and 53BP1 (green, **E**) in GSC19 transfected with shMYC or Control, including quantifications of positive staining cells (mean ± SD, images n =5, from 5 biologically independent samples).

F, Nude mice (nu/nu, n = 6) were intracerebrally implanted with either control GSCs or GSCs subjected to MYC knockdown (5×10^4 cells per mouse). The Kaplan–Meier curve illustrates the survival analysis for these cohorts, with statistical significance assessed using the Log-rank (Mantel-Cox) test.

G, The figure presents representative H&E stained sections of mouse brain tissue harvested 27 days after transplantation with GSCs. A scale bar indicating 1mm is provided for size reference.

H, A targeted metabolomic analysis was undertaken to discern the metabolic differences between control and MYC-knockdown GSC19.

I and **J**, IF staining for 8-OHdG (red, **I**) and 53BP1 (green, **J**) is demonstrated in GSC19-derived xenograft with either shMYC or Control transfection, including the quantification of positively stained cells (mean ± SD, images n =5, from 5 biologically independent samples).

K, IB confirms the restoration of PHGDH expression levels in GSC19 following MYC knockdown and subsequent re-overexpression of PHGDH.

L, The evaluation of peroxide production via H2-DCFDA fluorescence intensity in GSC19 shows that the re-overexpression of PHGDH partially reverses the effects of MYC knockdown (mean ± SD, n = 3 independent biological replicates).

M, IF staining demonstrates that re-overexpression of PHGDH in GSC19 attenuates the increase in the proportion of 53BP1 positive cells following MYC knockdown, suggesting a rescue of the DNA damage response (mean ± SD, n = 3 independent biological replicates).

Statistical analyses were performed using Welch’s two-sided t-test (B, C, H, L, M) and unpaired two-sided Student’s t-test (D, E, I, J).

Figure S7. PHGDH promotes GSC radioresistance through redox homeostasis and DNA repair

A-B, we evaluated the levels of cellular peroxides using H2-DCFDA fluorescence in GSC19 subjected to various treatments, including ionizing radiation (IR) at a dosage of 3 Gy over periods of 48 hours (**A**) or 72 hours (**B**). These oxidative stress indicators were quantified via flow cytometry (mean ± SD, n = 3 independent biological replicates), and statistically analyzed with an unpaired two-sided Student's t-test.

C-D, IB analyses reveal the expression levels of PARP, cleaved-caspase 3, and PHGDH in GSC19 following the same treatment regimens as above, over 48 hours (**C**) or 72 hours (**D**).

E-F, Depict the rate of cell apoptosis in GSC19 as measured by flow cytometry post-treatment IR, 3Gy for 48 hours (**E**) or 72 hours (**F**).

G-H, Quantification of apoptotic cells from **E** and **F** (mean ± SD, n = 3 independent biological replicates). statistically analyzed with an unpaired two-sided Student's t-test.

I, IF staining for 53BP1 (green) was conducted on GSC19 under the same treatment conditions. The resulting images are shown on the left, with quantifications of 53BP1-positive cells provided on the right (mean ± SD, n = 5, biologically independent samples, analyzed using an unpaired two-sided Student's t-test). The scale bar indicates 70 μm.

J-K, mirror those in a-b, but with GSC29, evaluating peroxide levels after 48 hours (**J**) or 72 hours (**K**) of treatment.

L-M, display the protein levels detected by immunoblotting in GSC29, analogous to c-d, for the respective treatment periods.

N-O and **P-Q**, designed in congruence with E-F and G-H, documenting and quantifying apoptosis in GSC29.

R, IF staining for γH2AX (red) in GSC29 post-treatment. The representative images are showcased on the left, with a graphical representation of the quantified γH2AX positive cells on the right (mean ± SD, n = 5, biologically independent samples, analyzed with an unpaired two-sided Student's t-test). The scale bar is set to 70 μm for these images as well.

Figure S8. Treatment with the inhibitor NCT-503 mimics the effects of PHGDH deletion

A, Treatment of various GSC lines (GSC19, GSC29, 4121) with escalating doses of NCT-503 was conducted over a range of days. Cell viability was evaluated at predetermined time intervals (Mean ± SD, n=3, biologically independent experiments).

B, IF staining was executed to detect Olig2 (red) in GSC19 GSCs treated with varying concentrations of NCT-503. Images on the left exhibit the staining, while quantifications of positively stained cells are provided on the right (mean ± SD, images n =5, from 5 biologically independent samples). A scale bar of 70 μm is provided for scale.

C-D, Nude mice (nu/nu), either harboring GSC-derived orthotopic xenografts 9 days post-transplantation or without xenografts, were administered NCT-503 (10mg/kg) intraperitoneally. Subsequently, mice were perfused with PBS and euthanized. NCT-503 concentration in mouse brains was quantified by LC-MS/MS chromatography. Representative LC-MS/MS chromatograms of NCT-503 in mouse brains are displayed (**C**), with accompanying quantifications of concentration (**D**) (Mean ± SD, n=5, biologically independent samples).

E-G, Representative images of IF staining for Olig2 (**E**), 8-OHdG (**F**), and cleaved-caspase 3 (**G**) in GSC19-derived xenografts are depicted (left). Quantitative analysis of cells positive for staining is presented (right, mean ± SD, images n =5, from 5 biologically independent samples). Cell nuclei were counterstained with Hoechst (blue). The scale bars measure 70μm.

H-N, Nude mice (nu/nu) received either a control treatment or NCT-503 (10mg/kg, daily). The weight trajectory of the mice is plotted (**H**) (Mean ± SD, n=5, biologically independent mice). Representative IF staining images for Olig2 (**I**) and Cleaved-caspase 3 (**J**) in the subventricular zone (SVZ) of mouse brains are shown (left; Scale bars, 70μm, with Hoechst counterstaining for nuclei). Quantifications of positively stained cells are provided (**I, J**, right, mean ± SD, images n =5, from 5 biologically independent samples). H&E stained sections of mouse brain (**K**), liver (**L**), lung (**M**), and kidney (**N**) tissues are exhibited. Scale bars measure 50μm.

O, Control or NCT-503 treated GSC19 were subjected to either CM or medium +SG. Flow cytometry analysis of peroxide levels was conducted post-treatment, alongside a quantification of the ROS intensity (mean ± SD, n = 3 independent biological replicates).

P, IF staining of 53BP1 in control and NCT-503 treated GSC19 subjected to either CM or medium +SG, with a quantitative assessment of 53BP1 positive cells (mean ± SD, images n =5, from 5 biologically independent samples).

Statistical analyses were performed using two-way ANOVA (A, H), Welch’s two-sided t-test (b, d), and unpaired two-sided Student’s t-test (E-G, I-J, O-P).

Fig. S1 Characterization and validation of GSC19 and GSC29

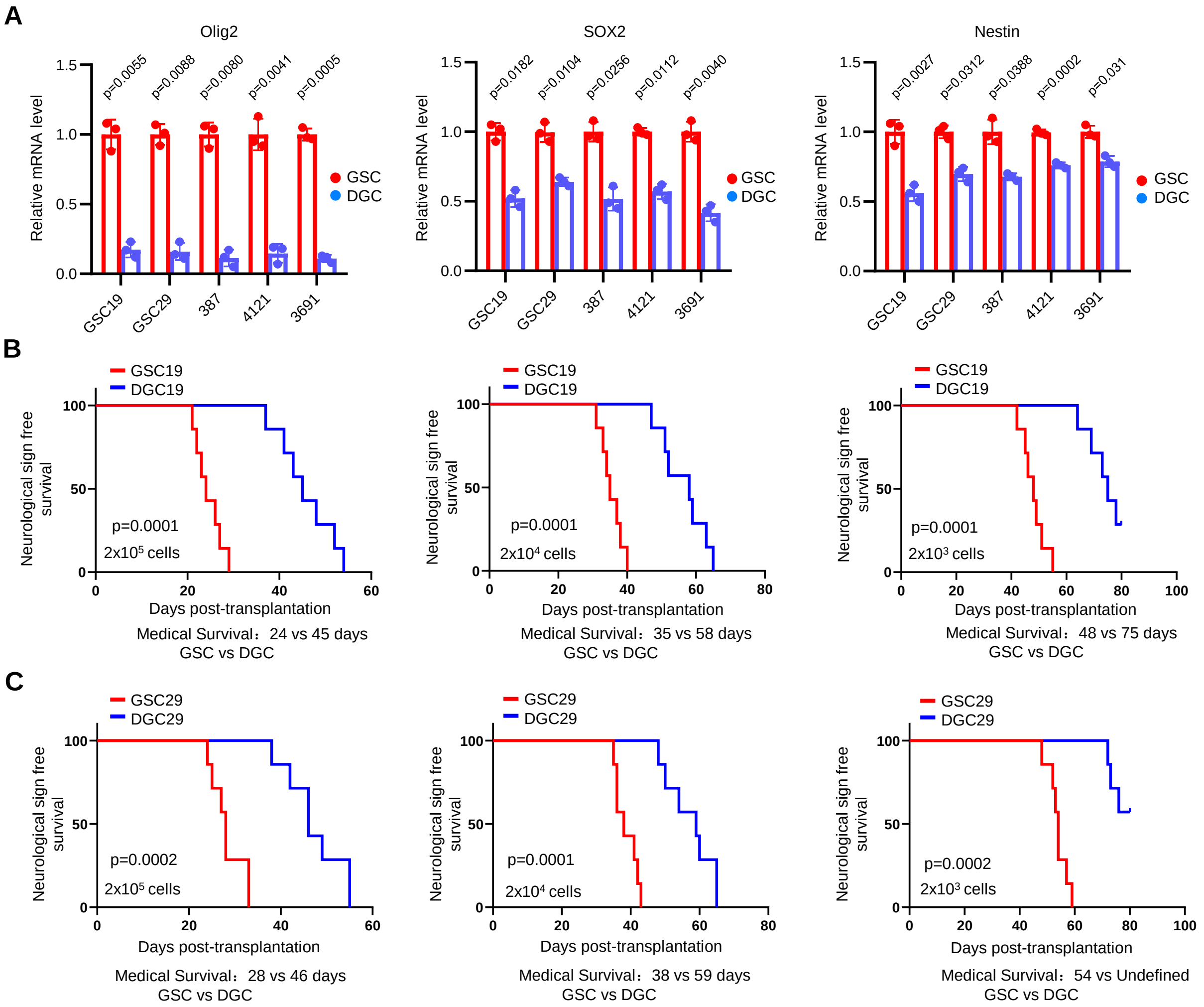

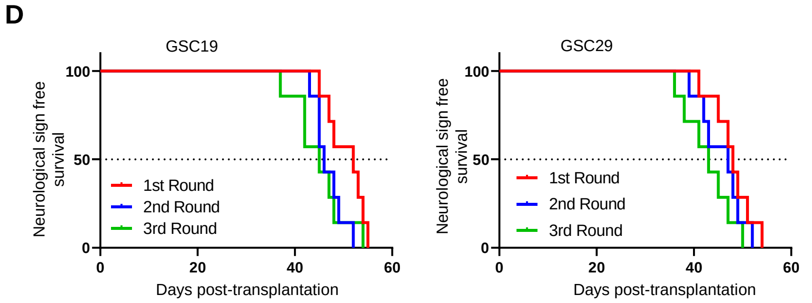

Demonstration of serial in vivo transplantation efficiency of GSCs

Series	Cell Name	Cell Number	Median Survival	Incidence
1st Round	GSC19	5000	52	7/7
	GSC29	5000	48	7/7
2nd Round	GSC19	5000	46	7/7
	GSC29	5000	47	7/7
3rd Round	GSC19	5000	45	7/7
	GSC29	5000	43	7/7

Fig. S2 PHGDH is strongly upregulated in GSCs

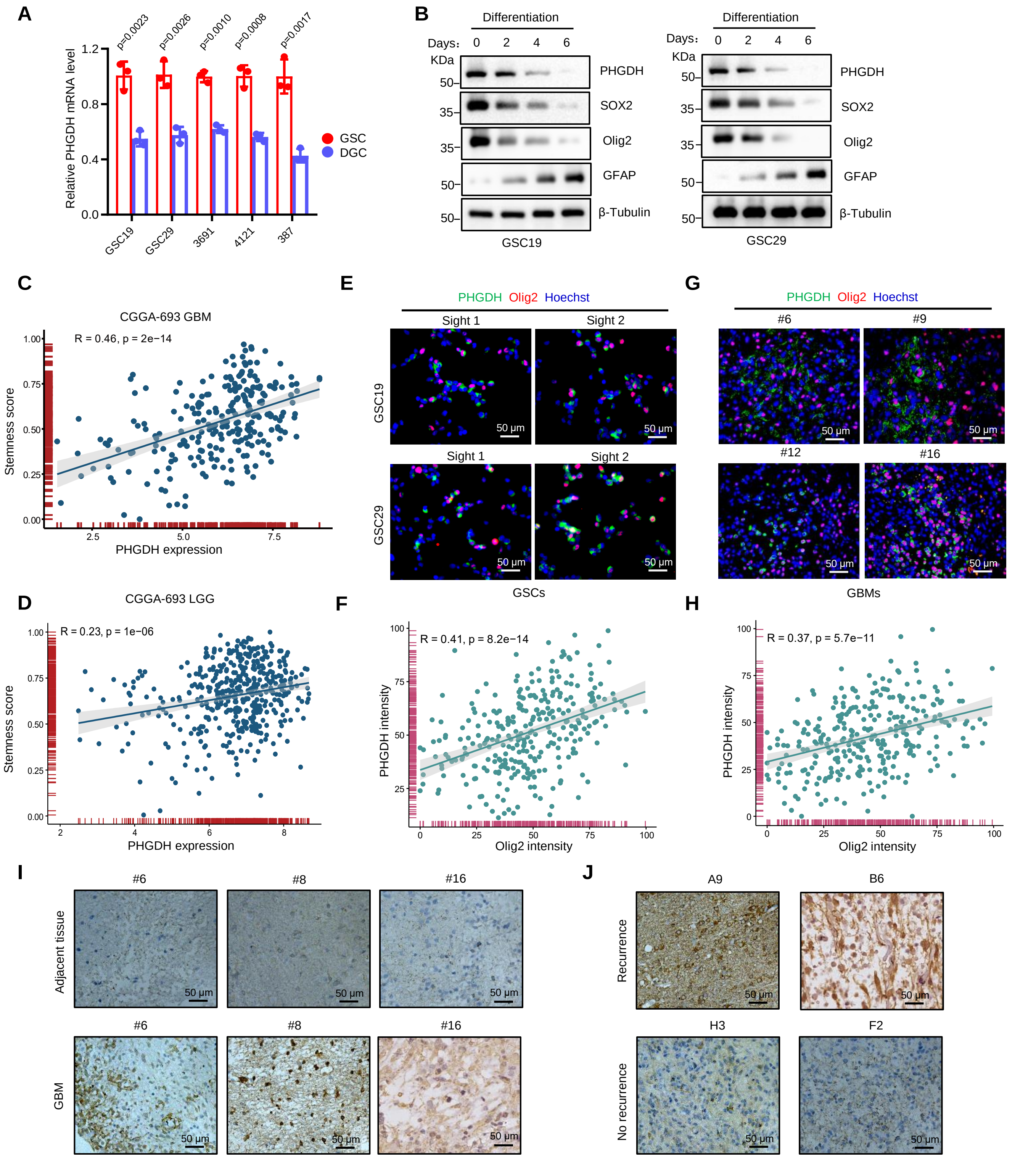

Fig. S3 PHGDH amplifies GSC self-renewal and accelerates tumor progression

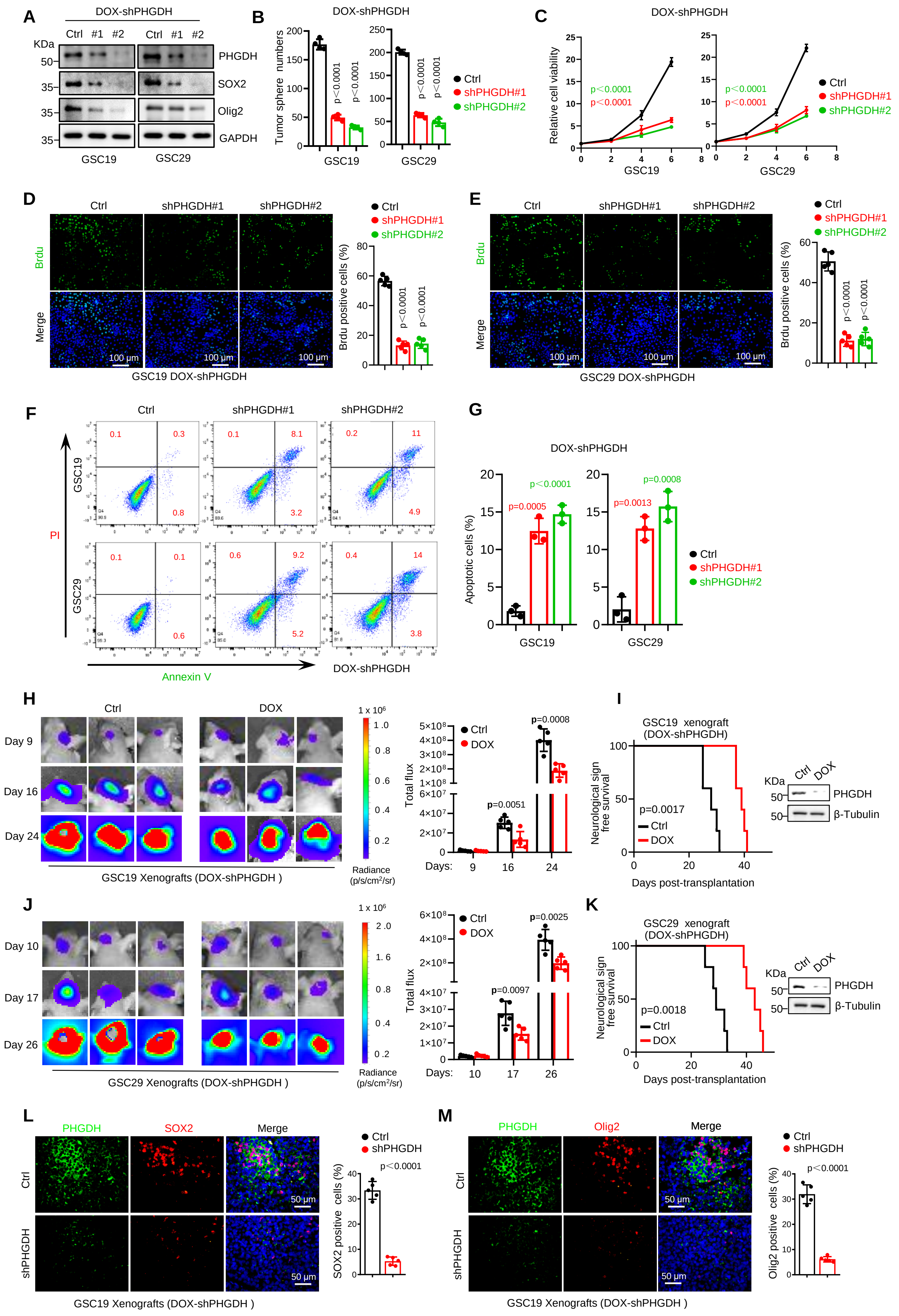

Fig. S4 PHGDH specifically maintains to redox homeostasis, one-carbon metabolism, and DNA repair through the SSP

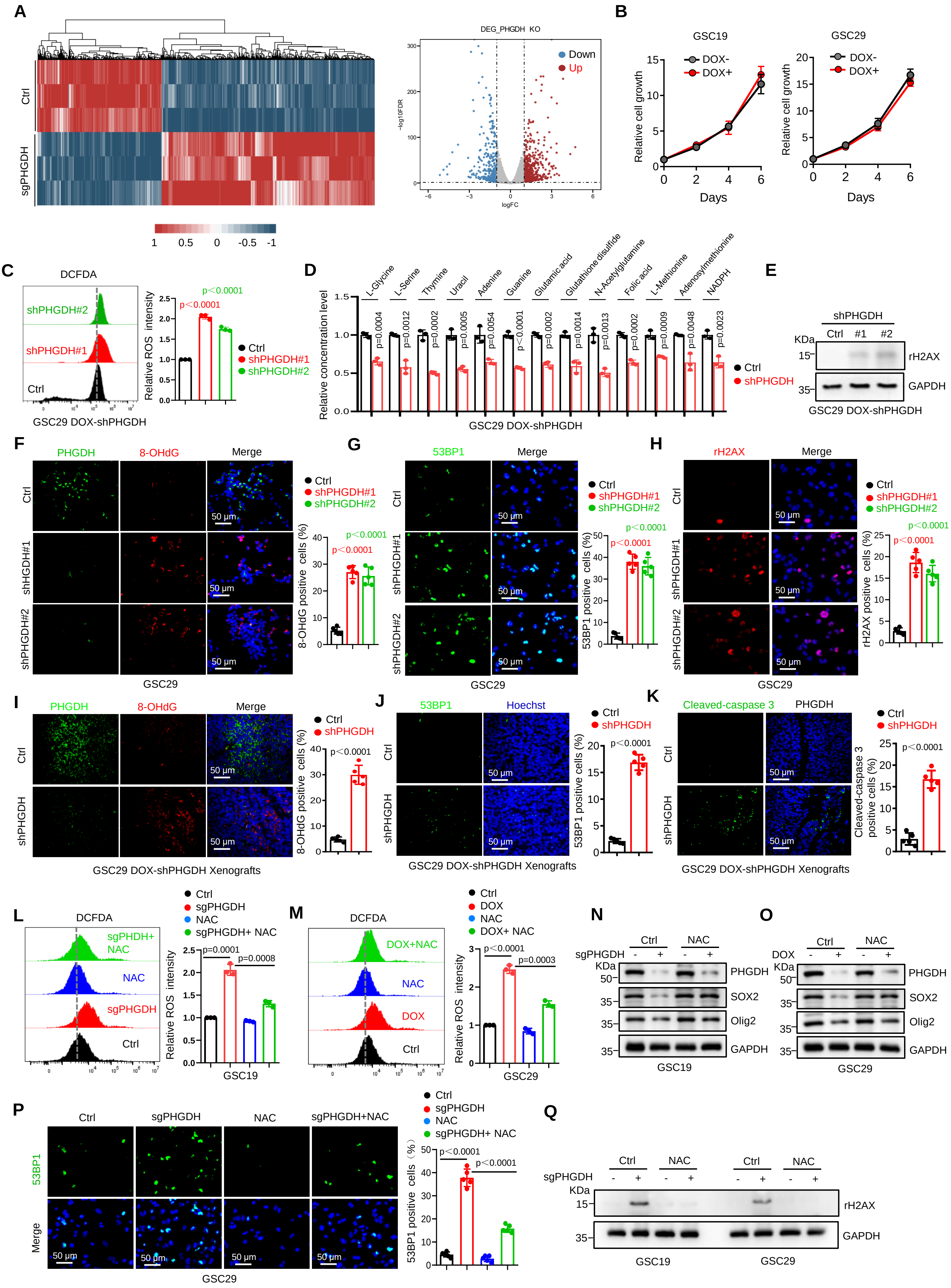

Fig. S5 Serine deprivation reveals the same effects to PHGDH deletion

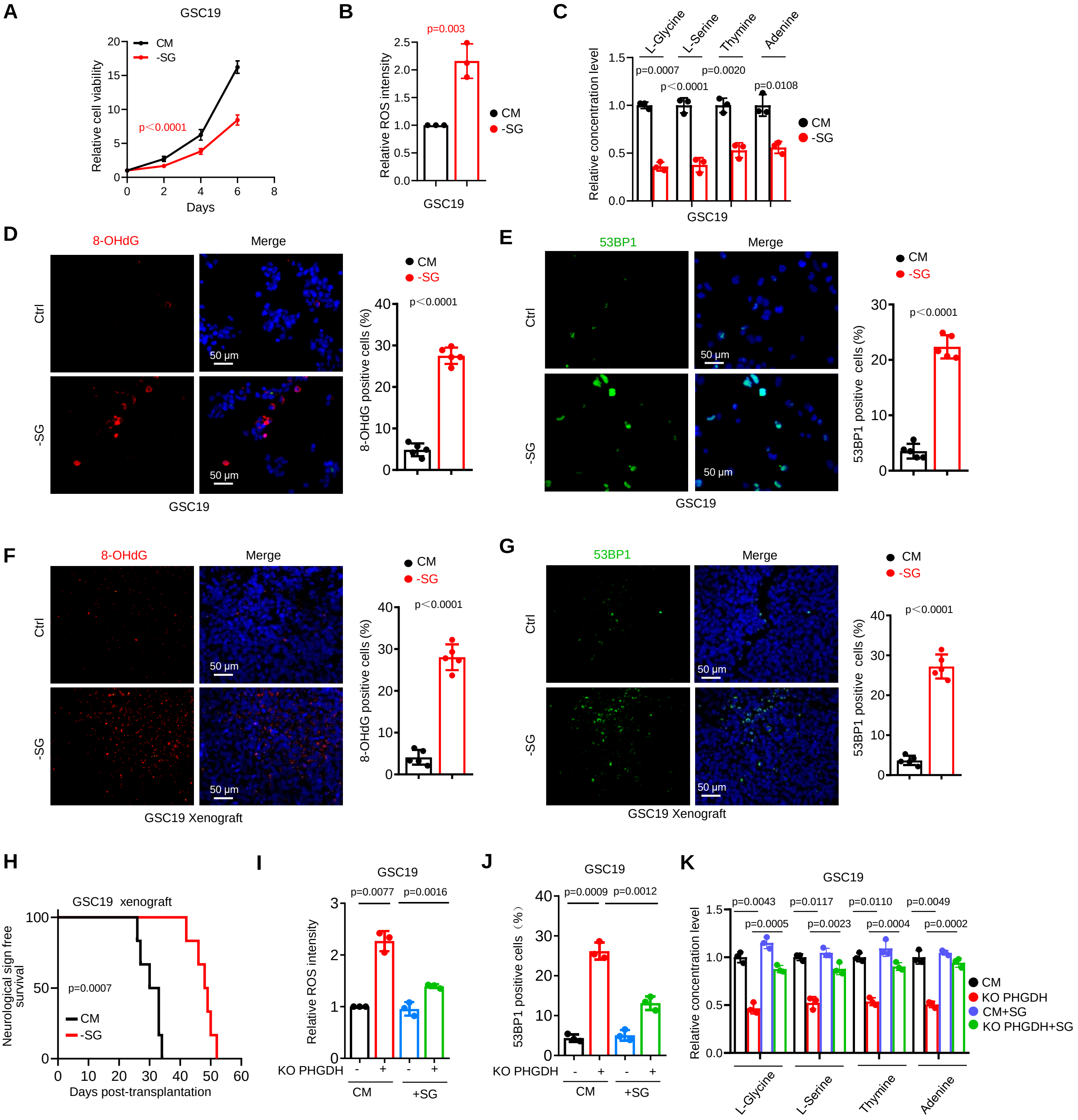

Fig. S6 MYC drives the expression of PHGDH in GSCs

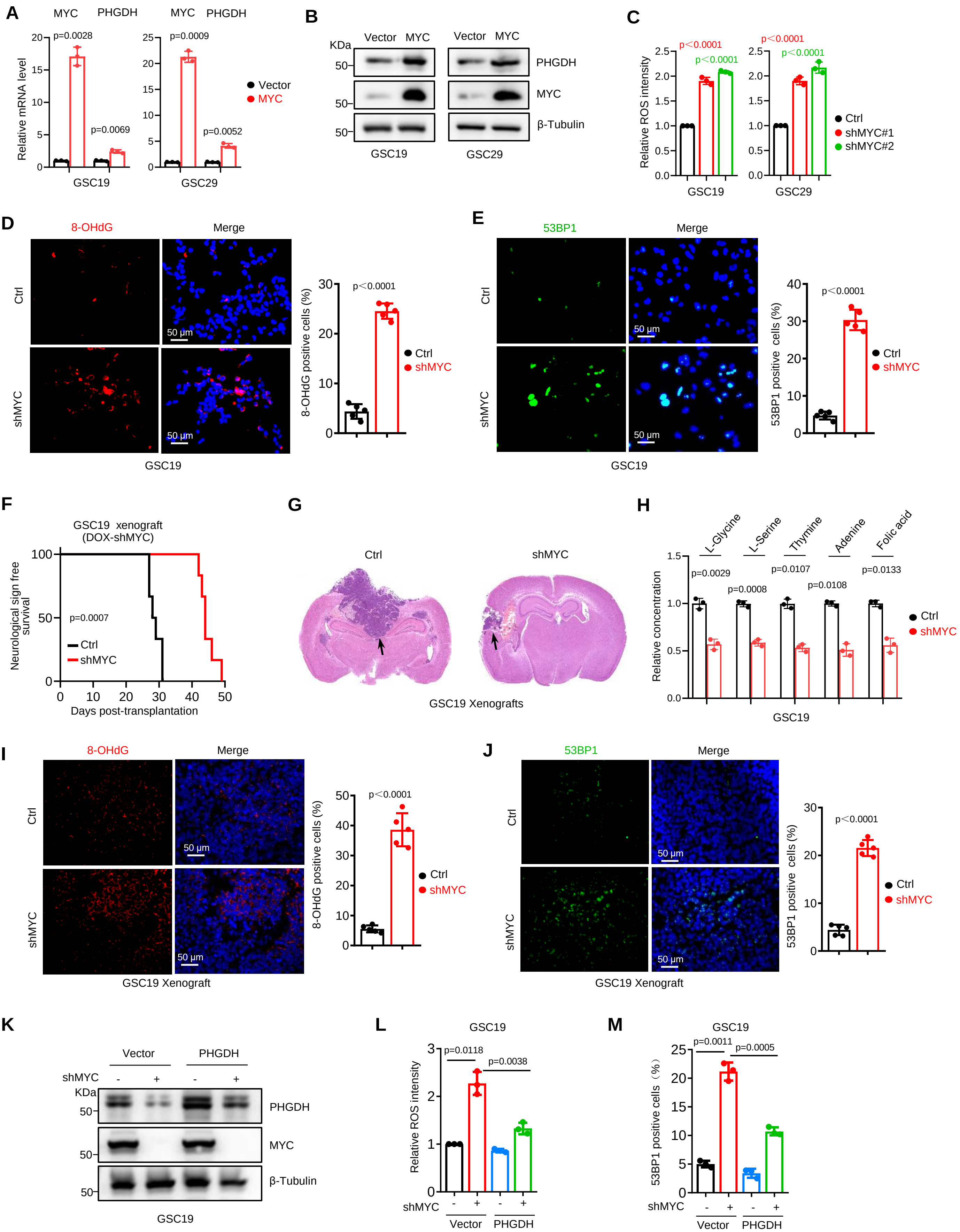

Fig. S7 PHGDH promotes GSC radioresistance through redox homeostasis and DNA repair

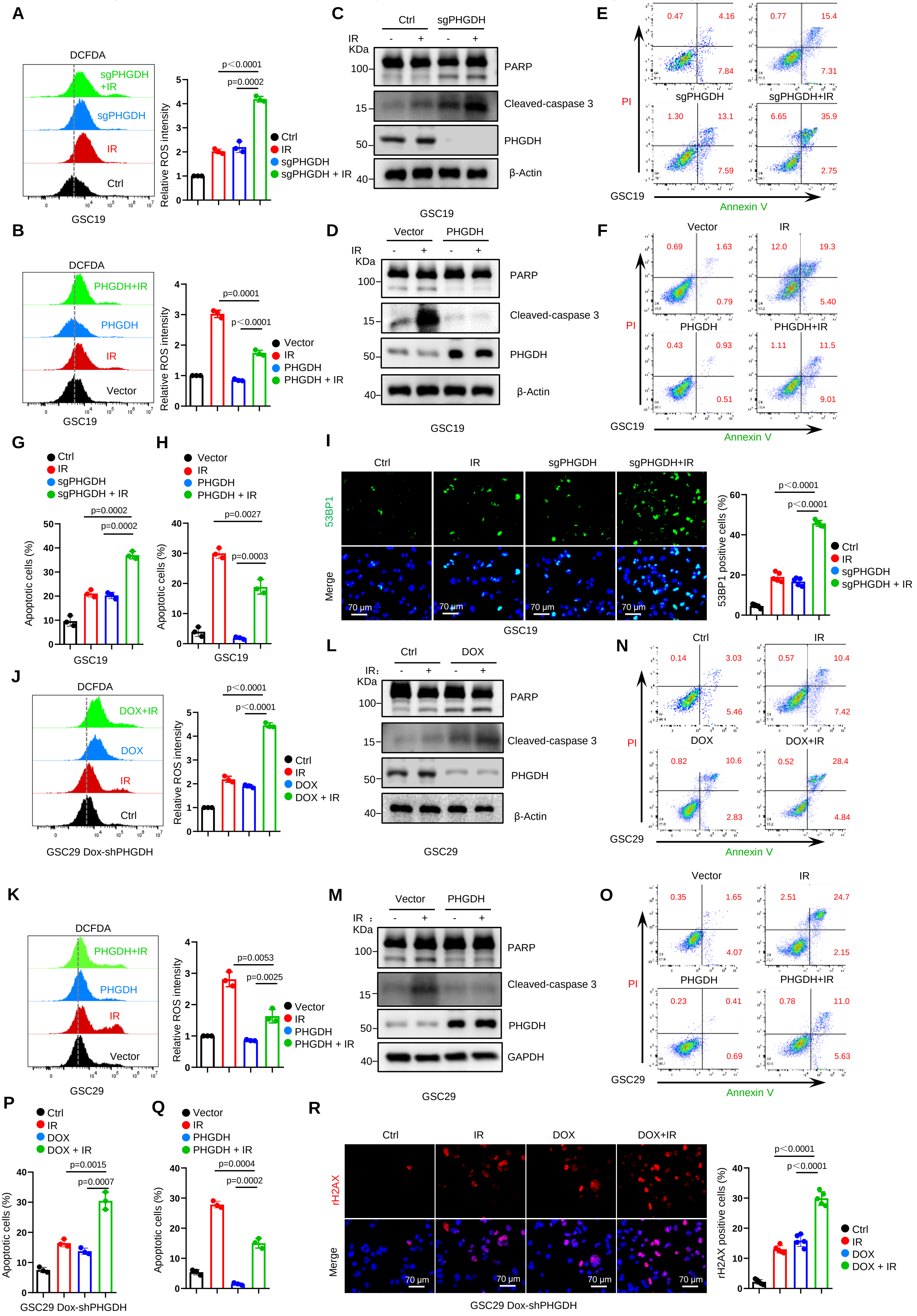

Fig. S8 Treatment with the inhibitor NCT-503 mimics the effects of PHGDH deletion

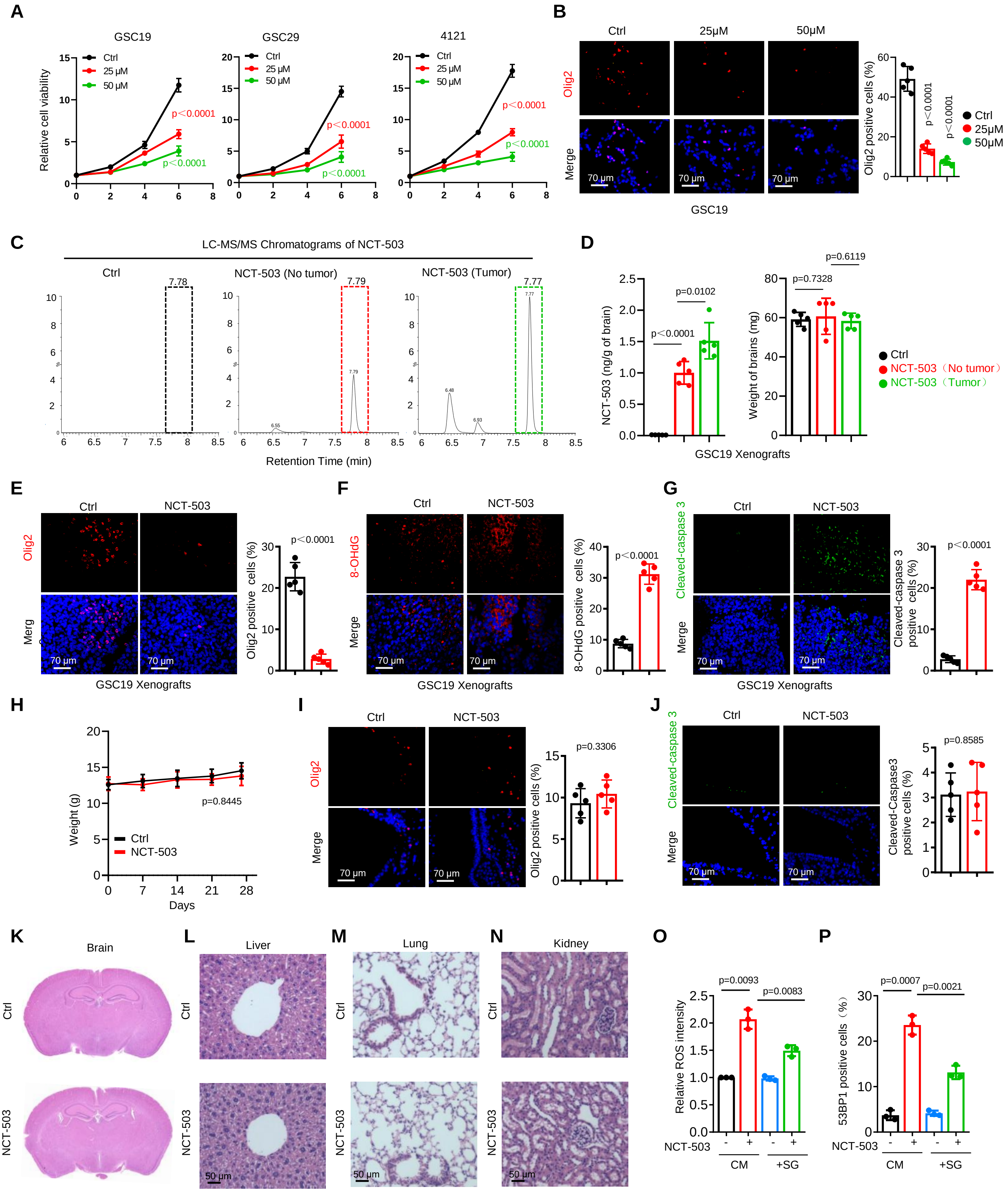

Supplementary Table 1

Expression of PHGDH and the pathological characteristics of human glioma patients of the Glioma Tissue Microarray.

Tissue identifier	Gender	Age (years)	WHO Grade	Recurrence	OS 0: alive; 1: dead	OS.time (months)	PHGDH intensity	PHGDH positive	PHGDH score
TJ01G10506	Male	39	II	N	0	114	2	20%	0.4
TJ01G10507	Male	22	I	N	0	114	1	70%	0.7
TJ01G10508	Female	46	II	N	0	67	2	10%	0.2
TJ01G10509	Female	44	II	N	0	113	2	60%	1.2
TJ01G10510	Male	65	II-III	N	1	32	3	90%	2.7
TJ01G10511	Female	71	II	N	1	48	1.5	100%	1.5
TJ01G10512	Male	47	I	N	0	112	3	60%	1.8
TJ01G10513	Female	18	I	N	0	111	1	50%	0.5
TJ01G10515	Female	19	IV	N	1	16	2	90%	1.8
TJ01G10516	Male	49	II	N	0	60	1	40%	0.4
TJ01G10517	Male	36	II	N	0	68	2	50%	1.0
TJ01G10518	Male	61	I	N	0	109	2	40%	0.8
TJ01G10519	Male	58	III	N	1	36	3	80%	2.4
TJ01G10521	Female	82	II	Recurred	0	109	2	80%	1.6
TJ01G10522	Male	30	II	N	0	66	2	70%	1.4
TJ01G10523	Female	64	III	N	0	32	2	80%	1.6
TJ01G10524	Male	68	II	N	1	84	1	60%	0.6
TJ01G10525	Male	57	II	N	1	32	2	50%	1.0
TJ01G10526	Male	48	II	N	0	65	1	50%	0.5
TJ01G10528	Male	31	III	N	0	38	2	80%	1.6
TJ01G10529	Male	38	II	N	0	57	2	50%	1.0
TJ01G10530	Male	51	II	N	0	54	2	40%	0.8
TJ01G10531	Male	63	II	N	0	106	2	5%	0.1
TJ01G10533	Female	38	II	N	1	52	2	40%	0.8
TJ01G10534	Male	41	II-III	N	0	73	2	40%	0.8
TJ01G10535	Female	58	I	N	0	105	2	50%	1.0
TJ01G10536	Female	20	IV	N	1	26	2	70%	1.4
TJ01G10537	Female	68	II	N	0	104	2	50%	1.0
TJ01G10538	Male	39	I	N	0	104	1.5	60%	0.9
TJ01G10539	Female	18	II	N	1	67	1	100%	1.0
TJ01G10541	Male	63	II	N	0	50	2	50%	1.0

TJ01G10542	Female	25	II	N	0	103	1	70%	0.7
TJ01G10543	Male	45	IV	N	1	18	3	90%	2.7
TJ01G10544	Female	59	I	N	0	102	3	50%	1.5
TJ01G10545	Female	34	II	N	0	64	1	70%	0.7
TJ01G10546	Male	57	III	Recurred	1	32	3	90%	2.7
TJ01G10547	Male	63	IV	N	1	8	3	80%	2.4
TJ01G10548	Female	36	I	N	0	101	2	80%	1.6
TJ01G10549	Male	39	III	N	1	40	2	80%	1.6
TJ01G10550	Female	46	II	Recurred	0	61	2	80%	1.6
TJ01G10551	Male	54	I	N	0	76	3	30%	0.9
TJ01G10552	Female	48	I	N	0	101	2	60%	1.2
TJ01G10553	Female	22	II	N	0	64	2	40%	0.8
TJ01G10554	Female	62	I	N	0	100	0	0%	0.0
TJ01G10555	Male	43	III	Recurred	0	54	2	50%	1.0
TJ01G10556	Female	53	II	N	0	103	1	80%	0.8
TJ01G10557	Male	43	II	N	0	100	2	10%	0.2
TJ01G10558	Female	70	III	N	0	40	2	70%	1.4
TJ01G10559	Male	33	II	Recurred	0	99	2	80%	1.6
TJ01G10560	Male	65	II	N	1	60	3	20%	0.6
TJ01G10561	Female	67	II	Recurred	0	99	3	30%	0.9
TJ01G10562	Male	59	III	Recurred	1	28	3	70%	2.1
TJ01G10563	Female	59	I	N	0	91	0	0%	0.0
TJ01G10565	Female	49	II	N	0	83	3	20%	0.6
TJ01G10566	Male	44	I	N	0	98	1.5	40%	0.6
TJ01G10568	Female	62	I	N	0	77	0	0%	0.0
TJ01G10569	Male	65	II	Recurred	0	97	3	30%	0.9
TJ01G10570	Female	22	II	N	0	92	2	70%	1.4
TJ01G10571	Female	71	II	Recurred	1	58	2	60%	1.2
TJ01G10572	Male	44	II	Recurred	0	97	1	100%	1.0
TJ01G10573	Male	28	IV	N	0	65	2	50%	1.0
TJ01G10574	Male	43	IV	Recurred	1	12	3	40%	1.2
TJ01G10575	Male	38	II	N	0	95	3	30%	0.9
TJ01G10576	Female	22	III	Recurred	0	42	3	80%	2.4
TJ01G10577	Female	38	I	N	0	94	1	70%	0.7
TJ01G10579	Male	20	II	N	0	53	0	0%	0.0
TJ01G10580	Male	44	III	N	0	34	3	90%	2.7

TJ01G10581	Female	43	II	Recurred	1	56	3	60%	1.8
TJ01G10582	Female	60	IV	Recurred	1	24	2	70%	1.4
TJ01G10583	Female	39	III	Recurred	0	56	2	50%	1.0
TJ01G10585	Female	37	IV	N	0	28	2	70%	1.4
TJ01G10586	Female	42	II	Recurred	0	93	2	70%	1.4
TJ01G10587	Female	48	II-III	Recurred	1	38	2	40%	0.8
TJ01G10588	Male	19	II	N	0	92	1	50%	0.5
TJ01G10589	Male	49	III	N	0	32	2	40%	0.8
TJ01G10590	Female	35	III	N	0	34	2	90%	1.8
TJ01G10594	Female	81	I	Recurred	1	34	3	50%	1.5
TJ01G10595	Female	76	II	Recurred	1	21	3	60%	1.8
TJ01G10596	Male	52	I	N	0	89	2	60%	1.2
TJ01G10597	Male	38	II	N	0	89	1	60%	0.6
TJ01G10598	Female	34	II	N	0	83	1	30%	0.3
TJ01G10599	Female	38	III	N	1	42	3	80%	2.4
TJ01G10600	Female	40	II	N	0	58	1	40%	0.4
TJ01G10601	Male	20	II	N	0	88	1	100%	1.0
TJ01G10602	Male	46	I	N	0	70	0	0%	0.0
TJ01G10603	Male	44	II	N	0	88	0	0%	0.0
TJ01G10604	Male	47	IV	N	1	10	3	80%	2.4
TJ01G10607	Female	70	II	N	1	63	3	10%	0.3
TJ01G10608	Male	35	III	Recurred	0	34	2	70%	1.4
TJ01G10609	Male	75	IV	N	1	22	2	60%	1.2
TJ01G10610	Male	30	II	N	0	87	2	5%	0.1
TJ01G10611	Male	39	II	N	0	92	2	20%	0.4
TJ01G10612	Male	71	II	N	1	43	3	10%	0.3
TJ01G10613	Female	38	II	N	0	86	1	30%	0.3
TJ01G10614	Male	51	I	N	0	91	1	40%	0.4
TJ01G10615	Female	43	II-III	N	1	33	3	20%	0.6
TJ01G10616	Female	21	II	N	1	74	1	40%	0.4
TJ01G10617	Female	61	III	N	0	82	2	50%	1.0
TJ01G10618	Female	43	II	N	0	85	2	30%	0.6
TJ01G10619	Female	60	III	N	1	20	3	80%	2.4
TJ01G10620	Male	42	II-III	Recurred	0	36	2	50%	1.0
TJ01G10621	Male	35	III	N	0	54	0	0%	0.0
TJ01G10622	Female	37	III	N	0	58	2	40%	0.8

TJ01G10623	Male	40	II	N	0	82	1	50%	0.5
TJ01G10624	Female	49	III	Recurred	1	64	3	30%	0.9
TJ01G10625	Female	36	IV	Recurred	1	42	3	30%	0.9
TJ01G10628	Male	60	IV	Recurred	0	25	3	40%	1.2
TJ01G10629	Male	32	III	Recurred	1	30	2	50%	1.0
TJ01G10630	Female	51	III	Recurred	1	36	2	70%	1.4
TJ01G10631	Male	50	II	Recurred	0	78	1	60%	0.6
TJ01G10633	Male	38	II	N	0	62	1.5	40%	0.6
TJ01G10634	Female	42	II	N	0	78	1	70%	0.7
TJ01G10635	Male	43	II	N	0	77	2	40%	0.8
TJ01G10636	Male	47	II	N	0	72	1	70%	0.7
TJ01G10637	Female	56	II	N	0	76	1	70%	0.7
TJ01G10638	Male	34	II	N	1	77	2	40%	0.8
TJ01G10639	Female	50	II	N	0	64	1	80%	0.8
TJ01G10641	Male	26	II	N	0	76	2	40%	0.8
TJ01G10642	Male	54	III	N	0	30	3	10%	0.3
TJ01G10643	Male	28	II-III	N	0	62	3	50%	1.5
TJ01G10644	Male	58	II	N	0	75	1	90%	0.9
TJ01G10645	Female	31	II	N	0	75	2	50%	1.0
TJ01G10646	Male	32	IV	N	0	56	2	10%	0.2
TJ01G10647	Male	72	III	Recurred	1	31	3	60%	1.8
TJ01G10649	Male	43	II	N	1	27	2	50%	1.0
TJ01G10650	Female	39	II	N	0	72	2	50%	1.0
TJ01G10651	Male	71	II-III	N	1	12	3	70%	2.1
TJ01G10652	Male	51	II	N	0	68	2	60%	1.2
TJ01G10653	Male	21	II	N	1	72	1	100%	1.0
TJ01G10654	Male	68	I	N	0	72	2	60%	1.2
TJ01G10655	Male	58	III	Recurred	1	26	3	80%	2.4
TJ01G10656	Male	22	II	N	0	71	2	60%	1.2
TJ01G10658	Female	59	III	N	0	35	3	60%	1.8
TJ01G10659	Female	45	I	N	0	70	2	60%	1.2
TJ01G10660	Male	38	III	N	0	30	3	60%	1.8
TJ01G10661	Female	42	III	Recurred	0	56	3	80%	2.4
TJ01G10663	Male	55	IV	N	1	22	3	60%	1.8
TJ01G10664	Male	26	IV	N	1	3	2	100%	2.0
TJ01G10665	Female	39	IV	Recurred	1	6	3	80%	2.4

TJ01G10666	Female	43	IV	N	0	26	2	80%	1.6
TJ01G10667	Female	46	IV	Recurred	1	16	3	50%	1.5
TJ01G10668	Male	24	IV	N	1	18	3	40%	1.2
TJ01G10669	Male	45	IV	N	1	16	3	70%	2.1
TJ01G10670	Female	62	IV	N	1	54	3	10%	0.3
TJ01G10671	Male	67	IV	Recurred	1	16	2	60%	1.2
TJ01G10672	Male	62	III	Recurred	1	42	3	40%	1.2
TJ01G10673	Female	58	IV	Recurred	1	18	3	90%	2.7
TJ01G10674	Male	48	IV	N	1	30	3	30%	0.9
TJ01G10676	Female	67	III	Recurred	0	32	3	40%	1.2
TJ01G10677	Female	73	IV	Recurred	1	24	2	60%	1.2
TJ01G10678	Female	43	IV	Recurred	0	50	2	70%	1.4
TJ01G10680	Female	80	IV	Recurred	0	38	2	50%	1.0
TJ01G10681	Female	64	IV	Recurred	1	14	3	60%	1.8
TJ01G10682	Female	69	III	Recurred	1	20	3	60%	1.8
TJ01G10683	Female	65	IV	Recurred	1	18	2	70%	1.4
TJ01G10684	Female	47	IV	Recurred	1	30	2	70%	1.4
TJ01G10685	Male	43	IV	Recurred	1	22	3	70%	2.1
TJ01G10687	Male	24	I	N	0	98	1	90%	0.9
TJ02G10003	Female	51	II-III	N	0	50	2	90%	1.8
TJ02G10004	Male	61	III	N	0	40	3	40%	1.2
TJ02G10006	Female	34	II	Recurred	0	89	2	50%	1.0
TJ02G10007	Female	60	III	Recurred	1	36	3	80%	2.4
TJ02G10009	Female	62	IV	Recurred	1	10	3	100%	3.0
TJ02G10010	Male	71	IV	N	1	18	3	90%	2.7
TJ02G10012	Male	68	II	Recurred	1	84	3	70%	2.1
TJ02G10013	Female	32	I	N	0	73	2	80%	1.6
TJ02G10014	Male	38	II	Recurred	1	56	3	50%	1.5
TJ02G10015	Male	19	II	N	1	48	2	90%	1.8
TJ02G10016	Female	56	II	N	0	103	2	60%	1.2
TJ02G10018	Male	51	II	Recurred	0	106	2	90%	1.8
TJ02G10020	Male	28	IV	N	1	28	3	90%	2.7
TJ02G10022	Female	46	I	N	0	114	2	70%	1.4
TJ02G10023	Male	50	II	Recurred	1	52	2	70%	1.4
TJ02G10025	Male	65	II	Recurred	0	97	3	60%	1.8
TJ02G10027	Female	43	II	N	0	85	2	70%	1.4

Supplementary Table 2. Primer sequences

The sequences of the sgRNAs and shRNAs

PHGDH-sgRNA#1	TTCGATGAAGGACGGCAAAT
PHGDH-sgRNA#2	AGCTGCGTTGATGACATCAG
PHGDH DOX-induced shRNA	CTTCGATGAAGGACGGCAAAT
PHGDH-shRNA#1	CAGGACTGTGAAGGCCTTATT
PHGDH-shRNA#2	CTTCGATGAAGGACGGCAAAT
MYC-shRNA#1	CCCAAGGTAGTTATCCTTAAA
MYC-shRNA#2	ACTGAAAGATTTAGCCATAAT

Primer sequences used for qRT-PCR analysis

MYC	5'-GGCTCCTGGCAAAAGGTCA-3'
	5'-CTGCGTAGTTGTGCTGATGT-3'
PHGDH	5'-CTGCGGAAAGTGCTCATCAGT-3'
	5'-TGGCAGAGCGAACAATAAGGC-3'
CDK4	5'-ATGGCTACCTCTCGATATGAGC-3'
	5'-CATTGGGGACTCTCACACTCT-3'
β-Actin	5'-AGAAAATCTGGCACCACACC-3'
	5'-AGAGGCGTACAGGGATAGCA-3'
GAPDH	5'-CCAGGTGGTCTCCTCTGACTTC-3'
	5'-GTGGTCGTTGAGGGCAATG-3'

Primer sequences used for Chip

PHGDH Forward	5'-GTCGAATTCCCTTCAACGTC-3'
PHGDH Reverse	5'-CCTGGTGAGCATATAAAAAGC-3'
GAPDH Forward	5'-TACTAGCGGTTTTACGGGCG-3'
GAPDH Reverse	5'-TCGAACAGGAGGAGCAGAGAGCGA-3'

Supplementary Table 3. Antibodies and agents

Primary antibodies

Names	Company	RRID
PHGDH	Proteintech (14719-1-AP)	AB_2283938
MYC	R&D Systems (AF3696)	AB_2282405
Caspase3/p17/p19	Proteintech (19677-1-AP)	AB_10733244
PARP	Proteintech (13371-1-AP)	AB_2160459
GAPDH	Proteintech (60004-1-Ig)	AB_2107436
β-actin	Proteintech (66009-1-Ig)	AB_2687938
β-tublin	Proteintech (10094-1-AP)	AB_2210695
Olig2	R&D Systems (AF2418)	AB_2157554
SOX2	R&D Systems (AF2018)	AB_355110
Anti-phospho-Histone H2A.X(Ser139)	Millipore(05-636-25UG)	AB_309864
Anti-DNA/RNA Damage(15A3)	Abcam (ab62623)	AB_940049
GFAP	Abcam (ab53554)	AB_880202
53BP1	Abclonal (A3859)	/

Secondary antibodies

Names	company	RRID
488-conjugated Goat anti-mouse	Invitrogen(A21202)	AB_141607
488-conjugated Donkey anti-rabbit	Invitrogen(A21206)	AB_2535792
Alexa Fluor™ 555-conjugated Donkey anti-Goat	Invitrogen(A21432)	AB_141788
Cy3-conjugated Goat anti-rabbit	Proteintech (SA00009-2)	AB_2890957
coralite 594, Goat Anti-Mouse	Proteintech (SA00013-3)	AB_2797133
Peroxidase-conjugated goat anti-rabbit	Proteintech (SA00001-2)	AB_2722564
Peroxidase-conjugated Goat anti-mouse	Proteintech (SA00001-1)	AB_2722565
Peroxidase-conjugated Donkey anti-goat	Proteintech (SA00001-3)	AB_2890882

Main agents

Names	company	Lot. No.
Doxycycline hydrochloride	MCE (HY-N0565A)	160296
Blasticidin A	MCE (HY-113542)	156353
NCT-503	MCE (HY-101966)	229651
